# An immediate transcriptional signature associated with response to the histone deacetylase inhibitor Givinostat in T acute lymphoblastic leukemia xenografts

**DOI:** 10.1038/cddis.2015.394

**Published:** 2016-01-14

**Authors:** M Pinazza, C Borga, V Agnusdei, S Minuzzo, G Fossati, M Paganin, B Michielotto, A De Paoli, G Basso, A Amadori, G te Kronnie, S Indraccolo

**Affiliations:** 1 Department of Surgery, Oncology and Gastroenterology, University of Padova, Padova, Italy; 2 Oncohematology Laboratory, Department of Woman and Child Health, University of Padova, Padova, Italy; 3 Immunology and Molecular Oncology Unit, Istituto Oncologico Veneto IRCCS, Padova, Italy; 4 Italfarmaco S.p.A, Milano, Italy; 5 Clinical Trials and Biostatistics Unit, Istituto Oncologico Veneto IRCCS, Padova, Italy

## Abstract

Despite some success with certain hematological malignancies and in contrast with the strong pro-apoptotic effects measured *in vitro*, the overall response rate of acute lymphoblastic leukemia (ALL) to histone deacetylase inhibitors (HDACis) is low. With the aim to improve the understanding of how HDACis work *in vivo*, we investigated the therapeutic efficacy of the clinically approved HDACi Givinostat in a collection of nine pediatric human T-ALL engrafted systemically in NOD/SCID mice. We observed highly heterogeneous antileukemia responses to Givinostat, associated with reduction of the percentage of infiltrating blasts in target organs, induction of apoptosis and differentiation. These effects were not associated with the T-ALL cytogenetic subgroup. Transcriptome analysis disclosed an immediate transcriptional signature enriched in genes involved in cell-cycle regulation and DNA repair, which was validated by quantitative RT-PCR and was associated with *in vivo* response to this HDACi. Increased phospho-H2AX levels, a marker of DNA damage, were measured in T-ALL cells from Givinostat responders. These results indicate that the induction of the DNA damage response could be an early biomarker of the therapeutic effects of Givinostat in T-ALL models. This information should be considered in the design of future clinical trials with HDACis in acute leukemia.

Histone deacetylases (HDACs) are enzymes involved in remodeling of chromatin and have a key role in the epigenetic regulation of gene expression. In recent years, inhibition of HDACs has emerged as a potential strategy to reverse aberrant epigenetic changes associated with cancer. HDAC inhibitors (HDACis) have various antitumor effects and have been shown to promote apoptosis, induce cell-cycle arrest and differentiation of tumor cells,^1, 2^ as well as to exert therapeutic activity in preclinical tumor models.^3, 4^ In patients, HDACis have demonstrated therapeutic potential for some hematological malignancies, including myelodysplastic syndromes, relapsed non-Hodgkin's lymphoma and mantle-cell lymphoma.^5^ Moreover, three HDACi (Vorinostat, Belinostat, Romidepsin) received FDA approval for cutaneous or peripheral T-cell lymphoma.^6^ Finally, FDA recently approved Panobinostat – a class I–II HDACi – for treatment of multiple myeloma in combination with Bortezomib and Dexamethasone.^7^

T-cell acute lymphoblastic leukemia (T-ALL) is a malignancy characterized by clonal expansion of T-lymphoid progenitors.^8^ Although the majority of pediatric T-ALL patients can be cured by current protocols, about one-fourth of patients has chemotherapy-resistant disease or relapse after therapy.^9^ Although these patients would greatly benefit from new treatments, the overall therapeutic potential of HDACis in acute leukemia is quite modest. In phase I clinical studies of Vorinostat and Tefinostat in patients with advanced leukemia or myelodysplastic syndrome, only a minority (<20%) of patients experienced hematological improvement or response.^10, 11, 12^ Future clinical trials with HDACis – either alone or in combination with other drugs – will likely require predictive biomarkers of response for patient stratification purposes.

In sharp contrast with the heterogeneous and often mild responses observed in patients, *in vitro* assays show substantially homogeneous and generally high cytotoxic responses of leukemia cells to HDACis.^3, 13, 14, 15^ What can account for this apparent discrepancy? In a recent preclinical study, it was shown that endothelial cells provide a Notch-dependent pro-tumoral niche for enhancing B-cell lymphoma survival and chemoresistance.^16^ Possibly, similar microenvironment-related mechanisms could contribute to attenuate the pro-apoptotic effects of HDACis, thus limiting therapeutic effects in some individuals.

Based on these considerations, when designing this study we considered mandatory to perform *in vivo* experiments with the final aim to better understand the cellular and transcriptional effects of HDACis in a complex leukemia model. We investigated antileukemia effects of Givinostat (ITF 2357), a pan-HDACi used in numerous phase II clinical trials, including for relapsed leukemias, myelomas^17^ and chronic myeloproliferative neoplasms,^18^ in patient-derived T-ALL xenografts. Heterogeneous antileukemia response to Givinostat were observed, and we found an immediate transcriptional signature enriched in genes involved in cell-cycle regulation and in DNA repair, which is associated with *in vivo* response to Givinostat.

## Results

### Therapeutic effects of Givinostat in T-ALL xenografts

To evaluate the therapeutic activity of HDACis in the contest of T-ALL, we initially set up a mouse trial with a panel of nine patient-derived xenografts, previously established from pediatric T-ALL samples in nonobese diabetic/severe combined immunodeficiency mice (NOD/SCID mice).^19^ Key clinical and genetic features of these xenografts and the donor's T-ALL, such as cytogenetic subgroup, prednisone sensitivity and MRD risk are reported in Table 1; the diagnostic immunophenotype is shown in Supplementary Table SIV.

**Figure 1:**
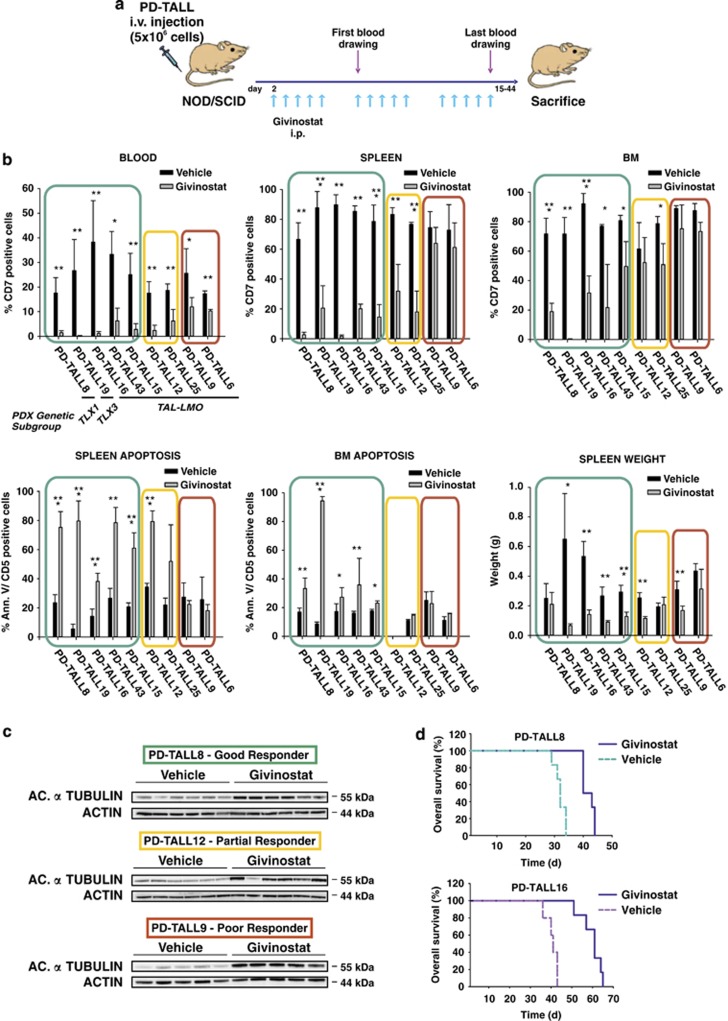
Therapeutic effects of Givinostat in patient-derived T-ALL xenografts. (**a**) Outline of treatment with Givinostat (ITF2357) or vehicle (PEG400/H_2_O). NOD/SCID mice (*n*=5/6 mice/group) were intraperitoneally treated with Givinostat (25 mg/kg) or vehicle 2 days after i.v. injection of T-ALL cells (5 × 10^6^cells/mouse). Givinostat was subsequently administered 5 days a week. Flow cytometric analysis of blood samples was used to track leukemia engraftment and progression. (**b**) Measurement of circulating blasts by flow cytometry after the last blood drawing (left panel, top) and quantification of infiltrating cells in the spleen (middle panel, top) and in the BM (right panel, top) at killing. Quantification of apoptotic leukemia cells in the spleen (left panel, bottom) and BM (middle panel, bottom). The spleen weight at killing was also reported (right panel, bottom). Results were expressed as mean value±S.D. Statistically significant differences are indicated (*P<0.05; **P<0.01; ***P<0.001). (**c**) Levels of acetylated *α*-tubulin were measured by western blotting analysis in PD-TALL8 (good responder), PD-TALL12 (partial responder) and PD-TALL9 (poor responder) cells obtained from the spleen of mice. A representative blot is shown. (**d**) Kaplan–Meier survival curves of mice engrafted with PD-TALL8 and PD-TALL16 after treatment with Givinostat or Vehicle (*n*=6 mice/group) (PD-TALL8: Log Rank *P*=0.0008; PD-TALL16: Log Rank *P*=0.0011)

In this early intervention trial, T-ALL cells were intravenously (i.v.) injected in NOD/SCID mice at 5 × 10^6^ cells/mouse (*n*=5/6 mice per group). Givinostat (25 mg/kg) or polyethylene glycol (PEG)400/H_2_O (vehicle) were administrated 5 days per week, and treatment started 2 days after cell injection and extended up to killing of the mice (Figure 1a).

Antileukemia response was evaluated by six parameters, including: (I) the percentage of CD7-positive cells in peripheral blood, (II–III) infiltration of leukemic cells in the spleen and bone marrow (BM) at killing, (IV–V) levels of apoptosis of CD5-positive cells in the spleen and BM at killing, and (VI) spleen weight. Xenografts were divided into good, partial and poor responders according to the modulation of at least five, two up to four and one parameters, respectively (Table 1).

PD-TALL8, PD-TALL15, PD-TALL16, PD-TALL19 and PD-TALL43 were good responders and displayed a significant reduction of leukemic cells in the blood, as well as in the spleen and BM at killing, and an increase in the levels of apoptosis in spleen and BM compared with controls (Figure 1b). Variations of spleen weight were detected only in some of the xenografts analyzed. PD-TALL12 and PD-TALL25 partially responded to treatment showing modulation of few parameters, including reduction of circulating cells and infiltration of spleen. Finally, PD-TALL6 and PD-TALL9 displayed minimal response to Givinostat, as shown by moderately reduced infiltration of spleen and BM by leukemic cells with minimal effects on T-ALL cell viability.

Despite markedly heterogeneous therapeutic effects, HDAC inhibition occurred in all samples, as shown by western blotting analysis of the acetylated form of *α*-tubulin in T-ALL cells from the spleen of mice representative of each group (Figure 1c). Notable, at variance with these *in vivo* results, incubation of Givinostat with T-ALL cells freshly isolated from the spleen of mice caused apoptosis in most leukemia cells (>80%), with minimal variations among the patient-derived xenograft (PDX) tested (data not shown).

We subsequently investigated whether HDAC inhibition could also improve survival. To this end, mice injected with PD-TALL8 and PD-TALL16 cells (*n*=6 mice per group) were treated by daily injections of Givinostat, starting 2 days after T-ALL cell injection. Compared with the control group, Givinostat extended survival of PD-TALL8 mice from 32±1.9 to 42±2 days (Log Rank *P*=0.0008) and survival of PD-TALL16 mice from 40±2.9 to 60±5 days (Log Rank *P*=0.0011; Figure 1d). In conclusion, these experiments indicated heterogeneous therapeutic effects of Givinostat in T-ALL xenografts, suggesting that intrinsic factors modulate therapeutic efficacy.

### Givinostat has mild effects on TLX and TAL-LMO target genes expression *in vivo*

**Figure 2:**
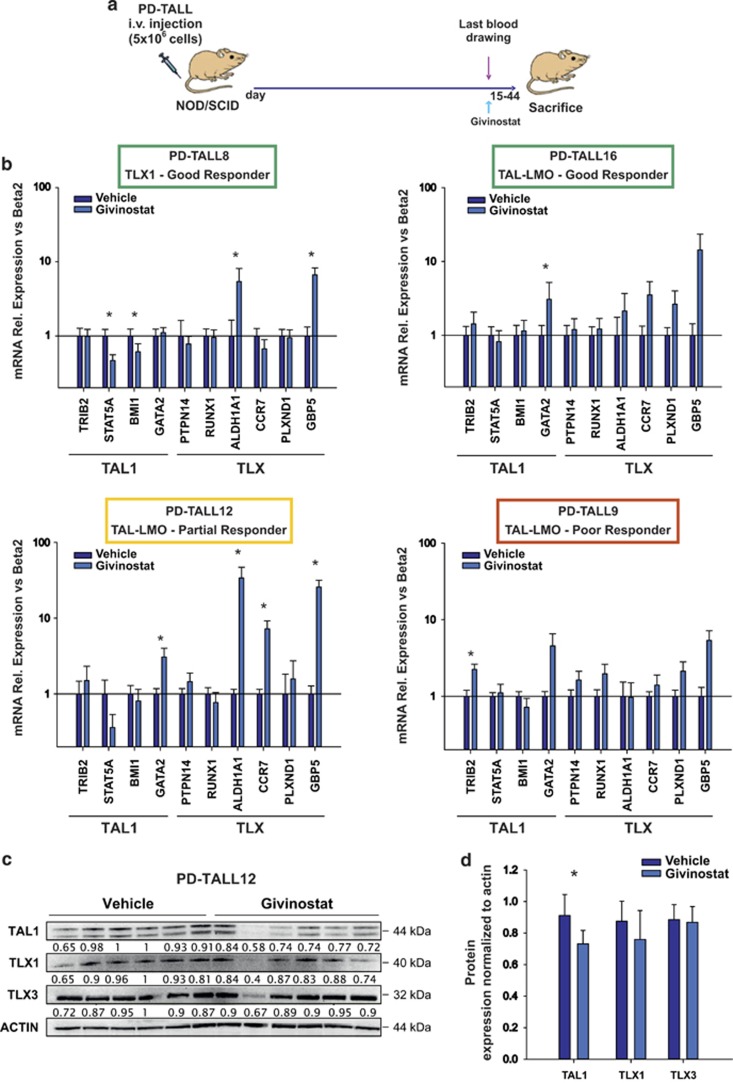
Expression levels of TAL1 and TLX target genes. (**a**) Outline of treatment. Leukemic NOD/SCID (*n*=5/6 mice/group) were intraperitoneally treated once with Givinostat (25 mg/kg) or vehicle. Mice were killed 6 h after treatment. (**b**) T-ALL cells were recovered from the mice spleen and mRNA expression of several target genes were assessed by qRT-PCR. Results were expressed as mean value±S.D. Data were analyzed with Mann–Whitney test with Bonferroni correction (*P<0.05). (**c**) Leukemic cells were recovered from the spleen of PD-TALL12 mice and TLX1, TLX3 and TAL1 protein levels were analyzed by western blotting. Numbers below the bands indicate densitometric analysis of TLX1, TLX3 and TAL1 normalized to ACTIN. (**d**) Columns report the mean values±S.D. of TLX1, TLX3 and TAL1 ratios in control and treated mice (*P<0.05)

Previous studies suggested that pro-apoptotic effects of HDACis in T-ALL cells could be due to downmodulation of TAL1 expression.^20^ To investigate whether antitumor effects were associated with relevant modulation of TAL-LMO signaling in our model, we treated NOD/SCID mice (*n*=5/6 per group) with Givinostat (25 mg/kg) or PEG400/H_2_O (vehicle). The drug was administered as a single dose when mice had full-blown leukemia – meaning the percentage of circulating blasts was >10% and the percentage of leukemic infiltrating cells in the spleen and BM was >85%. Spleen and BM infiltration by T-ALL cells was very high and comparable between treated and untreated mice (data not shown). Mice were killed 6 h after treatment (Figure 2a). We chose this time point based on previous experiments showing increased tubulin acetylation after 6 h of treatment with Givinostat (data not shown). Givinostat affected the expression of some TAL1 target genes (including STAT5A and BMI1), although these effects were not shared by all the xenografts tested and did not match antitumor responses (Figure 2b). Moreover, as TLX1 and TLX3 act as transcriptional repressors by forming a complex with HDACs,^21^ we investigated by quantitative RT-PCR whether HDACis could modulate the expression of TLX target genes. Interestingly, the expression of ALDH1A1, GBP5 and CCR7 was upmodulated in Givinostat-treated mice, suggesting attenuation of TLX-mediated transcriptional repression of these genes in some but not all xenografts (Figure 2b). Interestingly, Givinostat significantly reduced the protein levels of TAL1 *in vitro*, as previously found by Cardoso *et al.*,^20^ whereas TLX1 and TLX3 protein levels were not affected by HDAC inhibition (Figures 2c and d and Supplementary Figure S1), suggesting that Givinostat regulates transcriptional activity at promoter sites of TLX target genes.

Altogether, these findings indicated that Givinostat is associated with partial *in vivo* modulation of TLX and TAL1 signaling pathways in T-ALL cells. These effects, however, are not prominent and do not likely account for the therapeutic activity of Givinostat in mice.

### HDACis induced differentiation of a TLX1 xenograft *in vivo*

**Figure 3:**
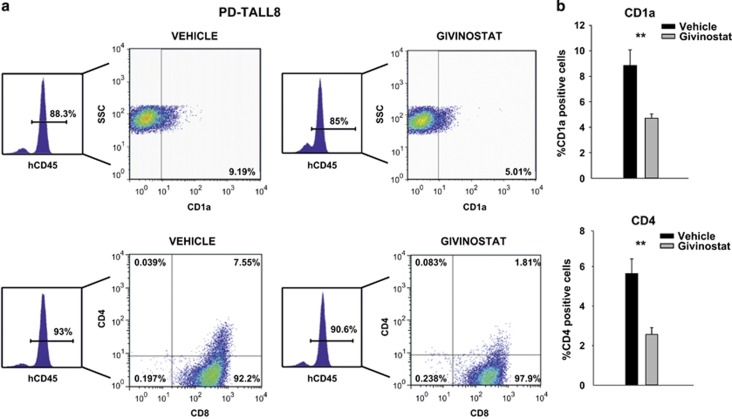
Givinostat induced differentiation of a TLX1-driven xenograft. (**a**) Flow cytometry analysis of CD1a (top) and CD4/CD8 (bottom) expression in human CD45-positive spleen cells isolated from PD-TALL8 leukemia recipient mice treated with Givinostat or vehicle (*n*=8/9 mice/group) for 5 days. A representative flow cytometry plot is shown. (**b**) Histograms report the mean values±S.D. of CD1a (top) and CD4 (bottom) in all mice analyzed. Data were analyzed with Mann–Whitney test with Bonferroni correction (**P<0.01)

As TLX1/TLX3 are well-established transcriptional repressors of differentiation^21, 22^ and some TLX-target genes were upmodulated by Givinostat *in vivo*, we next investigated whether Givinostat may restore cell differentiation. To this aim, we injected PD-TALL8 (TLX1) in NOD/SCID mice (*n*=8/9 mice per group), and when mice developed full-blown leukemia (as defined above), they were treated for 5 consecutive days with Givinostat or vehicle. We analyzed a panel of 13 T-cell surface markers, including CD1a, CD2, CD3, CyCD3, CD4, CD5, CD7, CD8, CD10, CD11b, CD34, CD99 and CD117. Treated mice displayed a significant reduction in the percentage of blasts expressing CD1a and CD4 surface markers and a slight, albeit not significant, reduction of the stem cell marker CD117 (Figure 3). These modulations involved markers of T-cell commitment (CD1a) and T-cell maturation (CD4). At the same time, treatment decreased the CD4^+^/CD8^+^ double-positive population. In the same experiment, only minimal variations in the proliferation marker Ki67 were detected (Supplementary Figure S5). As control of differentiation, we analyzed the PD-TALL16 xenograft, which belongs to the TAL-LMO subgroup and is characterized by a T mature phenotype (Supplementary Table SIV). In line with the differentiated phenotype of this xenograft, CD1a and CD117 were not expressed by PD-TALL16 cells, and no modulation of these differentiation markers or other T-cell surface markers was observed upon Givinostat treatment (data not shown). These results indicate initial differentiation of a TLX1-driven xenograft (PD-TALL8) following Givinostat administration without relevant effects on cell proliferation, fitting with the upregulation of some TLX target genes measured by quantitative RT-PCR (qRT-PCR) analysis.

### Response to Givinostat is not associated with cytogenetic subgroups

Next we argued that genetic subsets of T-ALL with dis-regulated expression of specific transcription factors might be more vulnerable to HDACis. To test this hypothesis, we used oligonucleotide microarrays (Affymetrix HG U133 Plus 2.0 GeneChip) to analyze the global patterns of gene expression in the T-ALL xenografts used in this study and to classify samples into the four main cytogenetic subgroups described elsewhere.^23^ Based on this analysis, seven out of the nine T-ALL xenografts (77.7%) belonged to the TAL-LMO subgroup, whereas the two remaining xenografts belonged to either the TLX1 or the TLX3 subgroup (Table 1). This finding was expected, as TAL-LMO is the most represented subgroup of T-ALL.^24^ Good responders included xenografts belonging to either TLX1/TLX3 or the TAL1-LMO subgroups, whereas poor responders were exclusively allocated to the TAL-LMO subgroup. Although limited by the small number of xenografts analyzed, these results – fitting the overall mild effects of Givinostat on transcriptional signatures coordinated by these transcription factors (see above) – indicate that T-ALL genetic aberrations are not associated with response to Givinostat treatment.

### Microarray analysis highlights a signature associated with therapeutic response to Givinostat

**Figure 4:**
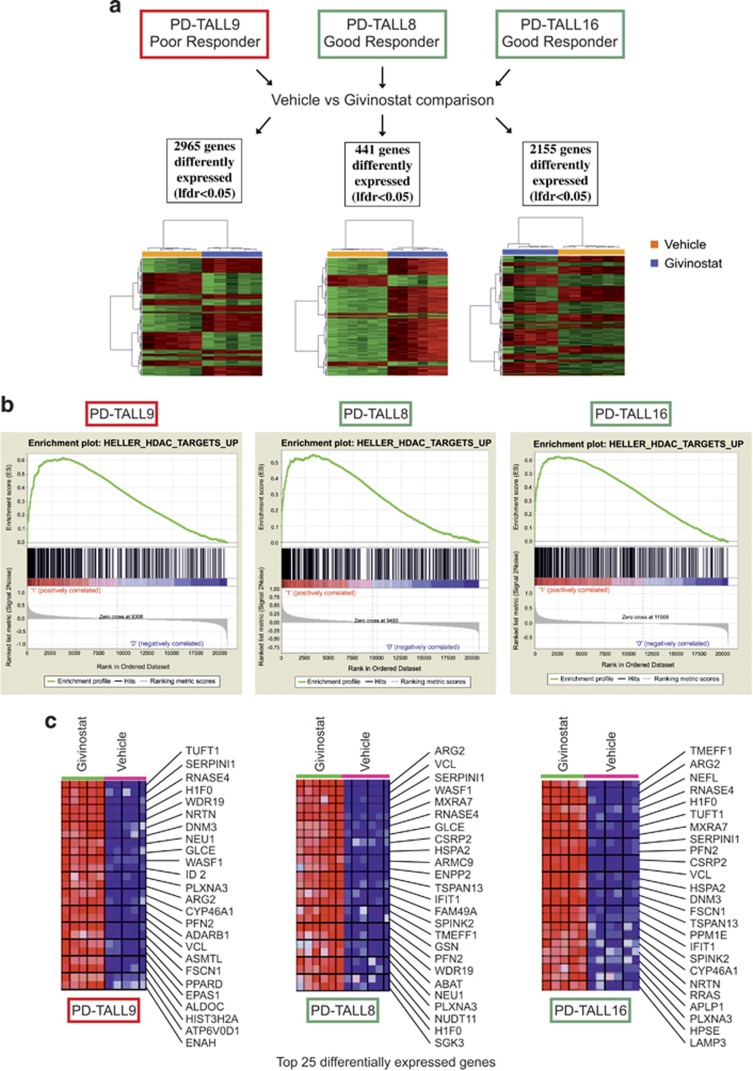
Positive enrichment of HDACi-related pathways in Givinostat-treated PDX by GSEA. (**a**) Heat maps depict for each set of xenografts supervised analysis of differentially expressed probes (LFDR<0.05) comparing Givinostat *versus* vehicle; PD-TALL9 (left), PD-TALL8 (middle) and PD-TALL16 (right) mice treated with Givinostat or vehicle for 6 h. The number of differentially expressed genes are reported. (**b**) GSEA plots of one of the top enrichment sets (HELLER_HDAC_UP) for PD-TALL9 (left), PD-TALL8 (middle) and PD-TALL16 (right) are shown. (**c**) Heat map representation of the top 25 differentially expressed genes in PD-TALL9 (left), PD-TALL8 (middle) and PD-TALL16 (right). The columns show individual samples. Red and blue indicate higher and lower expression levels, respectively

As the effects of Givinostat on specific transcription factors active in T-ALL cells did not seem likely to account for the marked antileukemia effects observed *in vivo*, to get a broader view of the transcriptional effects induced by Givinostat *in vivo*, we performed microarray analysis of T-ALL cells recovered from the spleen of Givinostat-treated mice and controls. To this end, we injected PD-TALL8 or PD-TALL16 cells (good responders) and PD-TALL9 (poor responder) in NOD/SCID mice (*n*=5/6 mice per group) and administered Givinostat or vehicle when mice had full-blown leukemia. Mice were killed 6 h later and oligonucleotide microarrays (Affymetrix HG U133 Plus 2.0 GeneChip) were used to analyze modulations of gene expression profiles induced by the drug. Shrinkage *t*-test, comparing the treated and vehicle groups for each set, revealed significant (local false discovery rate (LFDR)<0.05) differences in the expression of 2965, 441 and 2155 genes for PD-TALL9, PD-TALL8 and PD-TALL16, respectively. Heat maps depicting supervised analysis using these gene lists show the difference between treated and untreated groups for all sets analyzed (Figure 4a). Ingenuity Pathway Analysis (IPA), separately performed on genes with >1.2-fold change (log scale) for each set of treated *versus* vehicle comparison, revealed a significant repression of gene networks promoting cell survival and cell viability in the treated group of both good responders (*P*-value<0.004, *Z*-score<−3). On the contrary, these same pathways were predicted to be activated in the treated group of the poor responder (*P*-value<0.006, *Z*-score>2) (Supplementary Figure S2). These findings are in agreement with the data previously described, where high level of apoptosis were found in the spleen of good responders but not in the poor responder (Figure 1b). Interestingly, in all three sets of samples analyzed, Gene Set Enrichment Analysis (GSEA) showed a positive enrichment of several pathways related to HDAC inhibition for the treated group compared with the vehicle group. Enrichment plots and heat map representations of the top enrichment (HELLER_HDAC_UP) are shown in Figures 4b and c. This observation was in line with increased levels of acetylated tubulin *in vivo* (Figure 1c) and increased levels of acetylated histone 3 (lysine 9) in T-ALL cells treated *in vitro* with Givinostat (Supplementary Figure S3) and corroborates the observation that Givinostat inhibits HDAC activity both in poor and good responders. Microarray data further confirmed that at the basal level xenografts of good and poor responders did not show any difference in the expression of *SIRT2* and several *HDAC*s, including *HDAC6* (Supplementary Figure S4).

**Figure 5:**
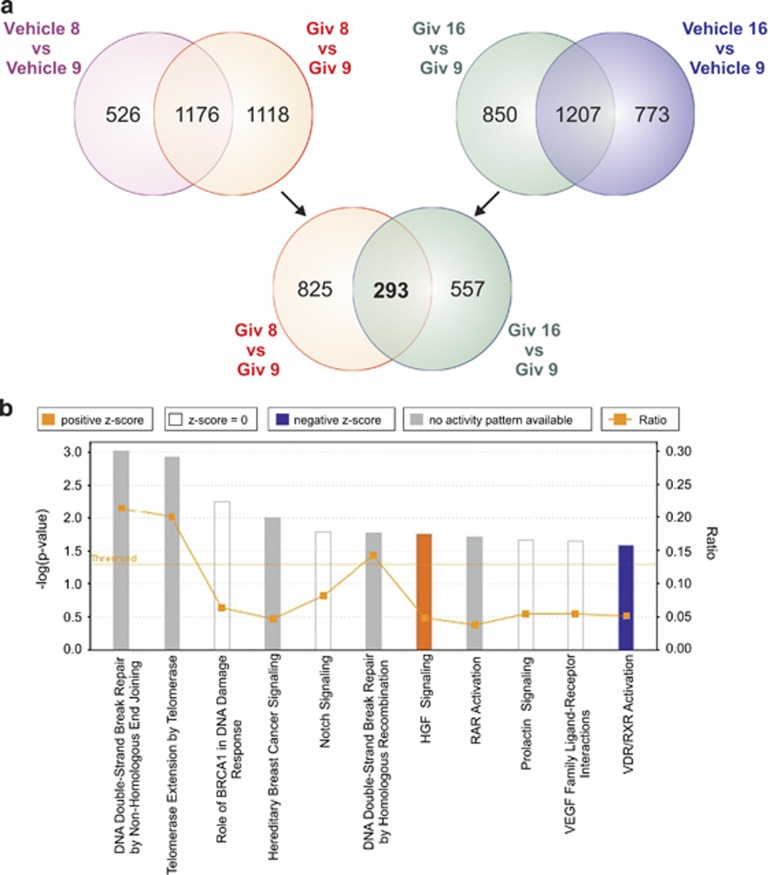
Identification of genes differentially regulated in good compared with poor responders upon Givinostat treatment and IPA analysis. (**a**) Venn diagram showing the common response (293 genes) to Givinostat treatment in both good responders (PD-TALL8 and PD-TALL16) compared with the poor responder (PD-TALL9). The list of 293 common genes results from the intersection between the genes specifically modulated by Givinostat treatment in each good responder compared with the poor responder (Giv8 *versus* Giv9 and Giv16 *versus* Giv9); genes that at the basal level are already differently expressed were removed for each set (Vehicle 8 *versus* Vehicle 9 and Vehicle 16 *versus* Vehicle 9). (**b**) Top canonical pathways for the list of 291 genes that characterized the good and poor response to Givinostat using IPA analysis. Results are scored based on the negative base 10 logarithm of the *P*-value (bars). The different color of the bars represent the predicted activation (*z*-score) for each canonical pathway. Orange lines: ratio, calculated as the ratio between the number of genes found in a pathway and the total number of genes that constitute that specific canonical pathway

**Figure 6:**
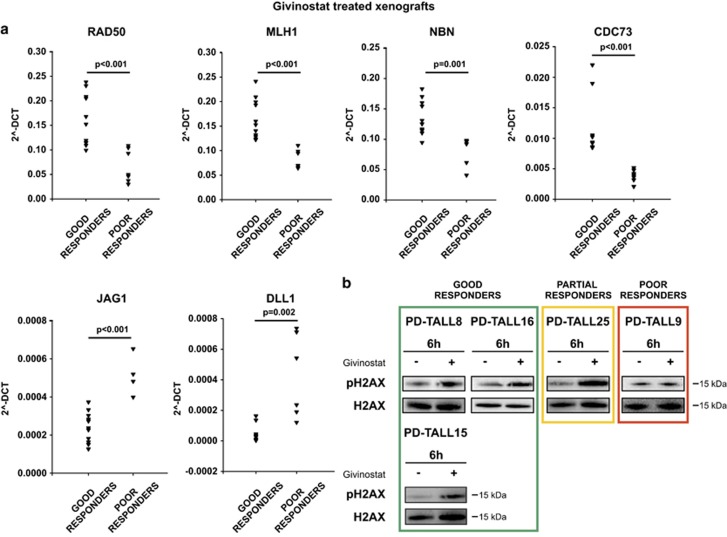
Good responders upregulated DNA repair genes compared with poor responders upon Givinostat treatment and increased DNA damage protein pH2AX. (**a**) *RAD50*, *MLH1*, *NBN*, *CDC73*, *JAG1* and *DLL1* expression analysis by qRT-PCR in good (PD-TALL8, PD-TALL16, PD-TALL43) and poor responders (PD-TALL9 and PD-TALL6) after 6 h of treatment with Givinostat *in vivo*. PD-TALL8 (*n*=2 mice), PD-TALL16 (*n*=5 mice), PD-TALL43 (*N*=4 mice), PD-TALL9 (*n*=4 mice), PD-TALL6 (*n*=3 mice). The 2^−Delta CT^ (Delta CT=CT gene–CT Beta2 microglobulin) was used as a read out of quantitative RT-PCR data. (**b**) Cells were recovered from the spleen of the xenografts and treated *in vitro* with Givinostat or vehicle for 6 h. pH2AX and total H2AX protein levels were then analyzed by western blotting

In order to retrieve the immediate response to 6-h Givinostat treatment independent from respective cytogenetic differences, the treated groups of good responders (Givinostat 8 and Givinostat 16) were disjointedly compared with the treated group of the poor responder (Givinostat 9). In addition, for each comparison, genes that were differentially expressed at the basal level were eliminated (comparison between Controls: Vehicle 8 *versus* Vehicle 9 and Vehicle 16 *versus* Vehicle 9, respectively). The intersection of the aforementioned comparisons identified 293 common genes of which 291 were upregulated (183 genes) or downregulated (108 genes) in both good responders compared with the poor responder (Figure 5a). The complete list of 291 genes is reported in Supplementary Table SV. The common behavior of 291/293 genes strongly suggests that the two good responders had a similar response to Givinostat, independently from their different cytogenetic background. Database for Annotation, Visualization and Integrated Discovery (DAVID) analysis on the list of the 291 common genes disclosed significant enrichment of genes related to the cell cycle (*P*-value=0.0004; Benjamin: 0.02), including several DNA repair-related genes in responsive xenografts (PD-TALL8 and PD-TALL16). IPA software revealed among the 291 common genes a significant enrichment of more than one pathway related to DNA repair in good responders compared with poor responder. Specifically, among the top canonical pathways, we found the DNA Double-Strand Break Repair by Non-Homologous End Joining (*P*-value: 9.77 E-04), Role of BRCA1 in DNA Damage Response (*P*-value: 5.53 E-03) and DNA Double-Strand Break Repair by Homologous Recombination (*P*-value: 1.7 E-02) (Figure 5b). Interestingly, all these DNA repair pathways had three genes in common: *RAD50*, *MLH*, and *NBN*. We validated these transcriptome findings by quantitative RT-PCR for samples used for microarray analysis and two additional PDXs treated with a single dose of Givinostat, including PD-TALL43 (good responder) and PD-TALL6 (poor responder). Results showed that poor responders displayed substantially lower expression levels of *RAD50, MLH* and *NBN* as well as the cell-cycle-related *CDC73* gene compared with good responders (Figure 6a). On the other side, we also analyzed the expression levels of *JAG1* and *DLL1*, 2 of the top 291 genes downregulated in good responders compared with poor responders (Figure 6a). As *RAD50, MLH1* and *NBN* are well-known DNA repair genes and we found them overexpressed in good responders, we checked protein levels of phospho histone 2AX (pH2AX), a marker of DNA damage. Interestingly, pH2AX levels increased both in good (PD-TALL8, PD-TALL16 and PD-TALL15) and partial responders (PD-TALL25) treated 6 h *in vitro* with Givinostat. On the contrary, pH2AX levels were not increased in the poor responder PD-TALL9 (Figure 6b). In conclusion, our results suggest that DNA damage response could be an early biomarker of the antileukemic effects of Givinostat in T-ALL models.

## Discussion

The PDX model is well established to investigate novel therapeutic approaches for T-ALL, as we and others have recently shown.^19, 25^ With regard to HDACis, Vilas-Zornoza *et al.*^3^ investigated the therapeutic effects of the LBH589 in ALL xenografts, but that study was limited to one T-ALL PDX and was therefore not adequately powered to detect possible variations in the magnitude of the therapeutic response among different PDXs. Here we evaluated the therapeutic activity of Givinostat, a pan-HDACi, in nine T-ALL PDXs. We observed dramatic differences in the therapeutic response, which enabled us to classify PDXs into good, partial and non-responders. Notably, increased acetylation of tubulin or histones was invariably observed, in line with previous clinical studies with other HDACis,^10^ indicating that Givinostat inhibited its pharmacological targets both in responders and non-responders. Moreover, no significant differences were observed in HDAC transcript levels (including HDAC1, HDAC3, HDAC5, HDAC6, HDAC8, HDAC10) among the various samples analyzed (data not shown). Induction of leukemia cell death was the most prominent biological effect of Givinostat *in vivo*. The percentage of apoptotic blasts in good responders was heterogeneous but generally higher in the spleen than in the BM (66.5±17.5% *versus* 42.7±29.8%). This finding resembles what we observed in a previous study with an antibody blocking the NOTCH ligand DLL4,^25^ probably reflecting a protective role of the BM microenvironment. In the case of PD-TALL8, we also found induction of cell differentiation, as indicated by variations in CD1a and CD4 expression levels (Figure 3). This result could be due to attenuation of TLX1/3 transcriptional repression activity, as suggested by increased levels of the TLX target gene *GBP5* measured in this PDX following Givinostat administration (Figure 2b). On the other hand, proliferation levels were barely altered, according to measurement of Ki67 positivity in PD-TALL8 samples (Supplementary Figure S5).

Gene expression profiling identified 291 genes differentially modulated by Givinostat in association with the therapeutic response. Among them, DAVID and IPA analysis identified a higher expression in good compared with poor responders of some genes involved in DNA repair and regulation of cell cycle, including *RAD50*, *MLH1*, *NBN* and *CDC73*, which were validated and extended by a qRT-PCR approach. RAD50 and NBN, together with MRE11, form a complex (also called MRN) critically important for chromosome stability for its role in repairing broken replication forks as well as two-ended double-strand breaks (DSBs) in both non-homologous end joining and homologous recombination repair pathways.^26^ The histone hyper-acetylation induced by HDACis causes structural alterations in chromatin, which may render DNA – normally protected by heterochromatin – more accessible to exogenous and endogenous DNA-damaging agents such as UV, X-ray, cytotoxic drugs or reactive oxygen species (ROS). In this regard, also Hu *et al.*^13^ measured increased levels of genes responsible for cellular defense against ROS (including GCLC, GSR, GST-pi and SOD1/2) following treatment of leukemia cells with Vorinostat. ROS could then be responsible for the induction of DNA damage response upon HDAC inhibition. Alternatively, it has been shown that chromatin remodeling can trigger DSBs sensing even before break recognition proteins binding to DNA ends.^26^ As certain HDACis can suppress DNA DSB proteins such as RAD50 protein,^27^ an higher amount of RAD50 transcripts, as well as other transcripts associated with DNA repair, upon Givinostat treatment, could be a compensatory response against oxidative stress.

In summary, we identified an immediate transcriptional signature, which is associated with response to Givinostat in T-ALL PDX. It is important to stress that in a previous retrospective analysis of a clinical study, upregulation of ROS scavengers appeared to be a mechanism of HDACi resistance.^10^ Moreover, in preclinical studies Vorinostat triggered ROS generation in HDACi-sensitive but not HDACi-resistant cells,^13^ and vorinostat-induced cytotoxicity was blocked by exposure to antioxidants.^15, 28^ Altogether, these observations hint at the possibility that Givinostat might cause stronger cytotoxic effects in leukemia cells endowed with high endogenous ROS levels. Indeed, responsive PDX increased pH2AX levels following Givinostat treatment *ex vivo*, whereas the poor responder PD-TALL9 displayed no variations in treated compared with untreated samples (Figure 6b). Interestingly, microarray data showed higher expression levels of several antioxidant genes (including *SOD2*, *TXN, GCLC, GCLX, RRM2B, BACH2* and *NFE2L2*) in the Givinostat poor responder compared with the two good responder PDXs (data not shown).

This notwithstanding, it cannot be ruled out that other mechanisms contribute to the antileukemia effect observed. For instance, we measured decreased Jagged-1 and DLL1 levels in leukemia cells from responsive PDX following Givinostat administration. In other experimental models, Jagged-1 contributes to stimulate NOTCH signaling and protect lymphoma cells from chemotherapy-induced apoptosis.^16^ Therefore, it could be that decreased Jagged-1 levels might attenuate NOTCH signaling in T-ALL cells. The role of Notch signaling in regulating T-ALL survival is well established,^19, 29^ but altogether our GEP data did not disclose reduced NOTCH signaling following Givinostat treatment, although we concede that impaired NOTCH signaling could emerge at later time points. Moreover, we found some evidence that Givinostat counteracts TAL1 signaling *in vivo*, as shown by reduction of TAL1 protein and STAT5 levels in some PDX (Figure 2b). The importance of TAL1 signaling in promoting T-ALL cell survival has been uncovered by others.^20^ Finally, blockade of TLX1/3 transcriptional repression activity could trigger T-ALL cells' differentiation, as discussed above. These findings are in agreement with numerous data showing a pro-differentiation effect of HDACis, a process well characterized in other leukemias, such as acute promyelocytic leukemia and acute myeloid leukemia.^30, 31, 32^ Still, none of these mechanisms seems to be the key driver of response *in vivo*, as therapeutic effects are not associated with a specific genetic subtype of T-ALL.

In conclusion, although our observations require further validation, such early response gene signature may enable future identification of patients who are more likely to benefit from treatment with Givinostat or possibly other clinically approved HDACis.

## Materials and Methods

### T-ALL xenografts' establishment and tumorigenicity assay

Primary T-ALL cells (PD-TALL) were obtained from the BM of newly diagnosed pediatric patients, according to the guidelines of the local ethics committees. Xenografts' establishment and their genetic characterization are reported elsewhere.^19^ NOD/SCID mice were purchased from Charles River (Wilmington, MA, USA). Procedures involving animals and their care conformed with institutional guidelines that comply with national and international laws and policies (EEC Council Directive 86/609, OJ L 358, 12 December 1987) and were authorized by the ethical committee of the University of Padova. Givinostat (ITF2357) was synthetized at Italfarmaco, Milan, Italy. Its purity and identity were confirmed by chromatographic and mass spectroscopic analyses. To test the therapeutic effects on leukemia cells, NOD/SCID mice were intraperitoneally injected with Givinostat (25 mg/kg) or PEG400/H_2_O (vehicle) 2 days after leukemic cells' injection. Givinostat was subsequently administered 5 days a week. Human CD5 and CD7, two surface markers highly expressed by T-ALL cells,^19^ were used to track leukemia engraftment by fluorescence activated cell sorting analysis. In all experiments, mice were inspected twice weekly to detect early signs and symptoms of leukemia, and blood was drawn to measure T-ALL cell engraftment. When the percentage of circulating human CD7-positive cells exceeded 15% (i.e., 15–44 days after cell injection, depending on xenograft), both groups were killed.

### Cytofluorimetric analysis

Anti-human FITC-conjugated CD5 and PE-Cy5-conjugated CD7 antibodies (Coulter, Fullerton, CA, USA) were used for the detection of T-ALL cells in blood and tissue samples. Apoptosis was evaluated using the Annexin-V-FLUOS Staining Kit (Roche Diagnostics, Penzberg, Germany). Antibodies utilized to analyze xenografts' immunophenotype are reported in Supplementary Table SI. Samples were analyzed on Beckman Coulter EPICS-XL Flow Cytometer (Coulter), BD LSRII Flow Cytometer or BD FACSCanto II (BD Biosciences, San Jose, CA, USA).

### Reverse transcription-PCR and quantitative PCR

Total RNA was isolated using TRIzol Reagent according to the manufacturer's instructions. cDNA was synthesized from 0.5 to 1 *μ*g of total RNA using the Super Script II Reverse Transcriptase Kit (Life Technologies, Paisley, UK). Expression levels of TAL1 and TLX target genes were analyzed by Real Time Ready custom panels (Roche Diagnostics), by using the δδCt method with normalization against *β*2-microglobulin expression. qRT-PCR analysis as validation of microarray results was performed using SYBR green (Life Technologies). Among the treated samples, we compared good responders with poor responders using the 2^−Delta CT^ (2^−^^CT gene^^−^^CT Beta2 microglobulin^) method. Primers used for qRT-PCR are reported in Supplementary Table SII.

### Western blotting analysis

Cells were re-suspended in lysis buffer (NP-40 1%, NaCl 150 mM, Tris HCl pH7.5 50 mM, EDTA 2 mM, NaF, Na_3_VO_4_ and protease inhibitor cocktail), and lysates obtained were quantified using Quantum protein Assay (EuroClone, Milan, Italy). About 30 *μ*g of proteins were denatured and loaded in a midi polyacrylamide gel 4–12% (Life Technologies). Separated proteins were transferred for 2 h at 400 mA on a nitrocellulose membrane (GE Health Care, Glattbrugg, Switzerland). Membranes were saturated ON at 4 °C with PBS–0.1% Tween–5% milk and then incubated with primary antibody according to the manufacturer's instructions. Immunoprobing was performed using the antibodies shown in Supplementary Table SIII and was followed by hybridization with a horseradish peroxidase-conjugated anti-rabbit or anti-mouse Ab (Perkin Elmer, Waltham, MA, USA). Antigens were identified by luminescent visualization using Western Lightning plus ECL reagents (Perkin Elmer).

### Gene expression profiling and classification of T-ALL xenografts

Total RNA from the spleen of individual xenografts was extracted using Trizol according to the manufacturer's instruction (Life Technologies). RNA concentration was determined using NanoDrop ND-1000 Spectrophotometer (NanoDrop Technologies Inc., Wilmington, DE, USA). RNA quality and purity control was assessed on the Agilent Bioanalyzer 2100 (Agilent Technologies, Waldbronn, Germany). Only RNA samples that passed these quality controls were used to perform microarray (Affymetrix HG U133 Plus 2.0 GeneChip Arrays, Affymetrix, Santa Clara, CA, USA) analysis. *In vitro* transcription, hybridization and biotin labeling were performed following the GeneChip 3'IVT Express Kit protocol (Affymetrix). Microarray data (.CEL files) were generated using the Affymetrix GeneChip Command Console Software (AGCC). All microarrays passed the quality controls: scale factor, number of present calls, internal probe calls, Poly-A controls, and the ratio GAPDH/β-actin 3′/5′. Microarray data (.CEL files) normalized using the justRMA algorithm were analyzed using R-Bioconductor (Version 2.15.3). Differentially expressed probe sets were identified by the Shrinkage *t*-test ^33^ and an LFDR was used to correct the *P*-value. For differently expressed probe sets between compared groups an LFDR <0.05 was considered significant. Differently expressed probe sets derived from the Shrinkage *t*-test were used for supervised analysis. Supervised classification (PAM (predictive analysis of microarrays)) was used to construct a predictive algorithm able to classify samples for the main cytogenetic subgroups (TAL/LMO, TLX1, TLX3, HOXA) as previously described.^23^ Microarray data have been deposited in NCBI's Gene Expression Omnibus (GEO; http://www.ncbi.nlm.nih.gov/geo/) and are accessible through GEO accession number GSE69346.

### Gene ontology analysis

The list of 291 common genes was analyzed for Gene ontology using DAVID v6.7.

### Ingenuity pathway analysis

Ingenuity Pathway Analysis 9.0 (Ingenuity Systems, www.Ingenuity.com) was used to perform a comprehensive analysis of treated/vehicle comparison for each set of xenografts and to analyze the list of 291 genes that characterized the good or poor response to Givinostat treatment. This analysis allowed to identify the most significant biological functions, gene networks and canonical pathways associated with these signature. The Core Analysis was used to compare our lists of genes with data from literature, and Fisher's exact test was used to perform the analysis.

### Gene set enrichment analysis

GSEA software version 4.0 was used to identify gene sets in the public domain that share the expression pattern found in the current study.^34^ For each group of gene sets, GSEA calculates and evaluates the statistical significance of an enrichment score (ES). The ES reflects the degree to which a gene set is overrepresented. GSEA analysis was performed, collapsing the probe sets to gene vectors and using the signal-to-noise metric, the gene-set permutation type and 1000 permutations. As recommended by GSEA guidelines, only gene sets with an FDR *q-*value<0.05 were considered.

### Statistical analysis

Results were expressed as mean value±S.D. Statistical analysis of data was performed using Student's *t*-test, when samples followed a normal distribution, or non-parametric Mann–Whitney test with Bonferroni correction when appropriate. Differences were considered statistically significant when *P*<0.05.

## Figures and Tables

**Table 1:** Clinical and molecular features of T-ALL patients and xenografts

**Sample ID**	**Gender**	**Age (years)**	**Phenotype**	**MRD risk**	**PGR/PPR**	**PDX genetic subgroup**	**PDX response to Givinostat**
PD-TALL6	M	13	T Int	MR	PGR	TAL-LMO	Poor
PD-TALL8	F	3	T Int	MR	PPR	TLX1	Good
PD-TALL9	M	9	Early T	HR	Deceased	TAL-LMO	Poor
PD-TALL12	M	4	Early T	MR	PGR	TAL-LMO	Partial
PD-TALL15	M	7	T	HR	PPR	TAL-LMO	Good
PD-TALL16	M	5	T Mat	MR	PPR	TAL-LMO	Good
PD-TALL19	M	16	Early T	MR	Relapse	TLX3	Good
PD-TALL25	M	9	T	SR	PPR	TAL-LMO	Partial
PD-TALL43	M	15	T Int.	MR	PGR	TAL-LMO	Good

Abbreviations: F, female; HR, high risk; M, male; MR, medium risk; MRD, minimal residual disease; PDX, patient-derived xenograft; PGR, prednisone good responder; PPR, prednisone poor responder; SR, standard risk.

## Abbreviations

BM: bone marrow
HDACi: histone deacetylase inhibitor
i.v.: intravenously
NOD/SCID mice: Nonobese diabetic/severe combined immunodeficiency mice
PDX: patient-derived xenograft
PEG: polyethylene glycol
pH2AX: phospho histone 2A.X
T-ALL: T-cell acute lymphoblastic leukemia.
