# Supplementary material for: An immediate transcriptional signature associated with response to the histone deacetylase inhibitor Givinostat in T acute lymphoblastic leukemia xenografts
Source: Cell Death Dis. 2016 Jan 14;7(1):e2047–. doi: 10.1038/cddis.2015.394 (PMC4816177; doi:10.1038/cddis.2015.394)
Supplement: Supplementary Information [file cddis2015394x1.docx]

**SUPPLEMENTARY DATA**

**Supplementary figures and tables**

**Suppl. Fig. 1**

**
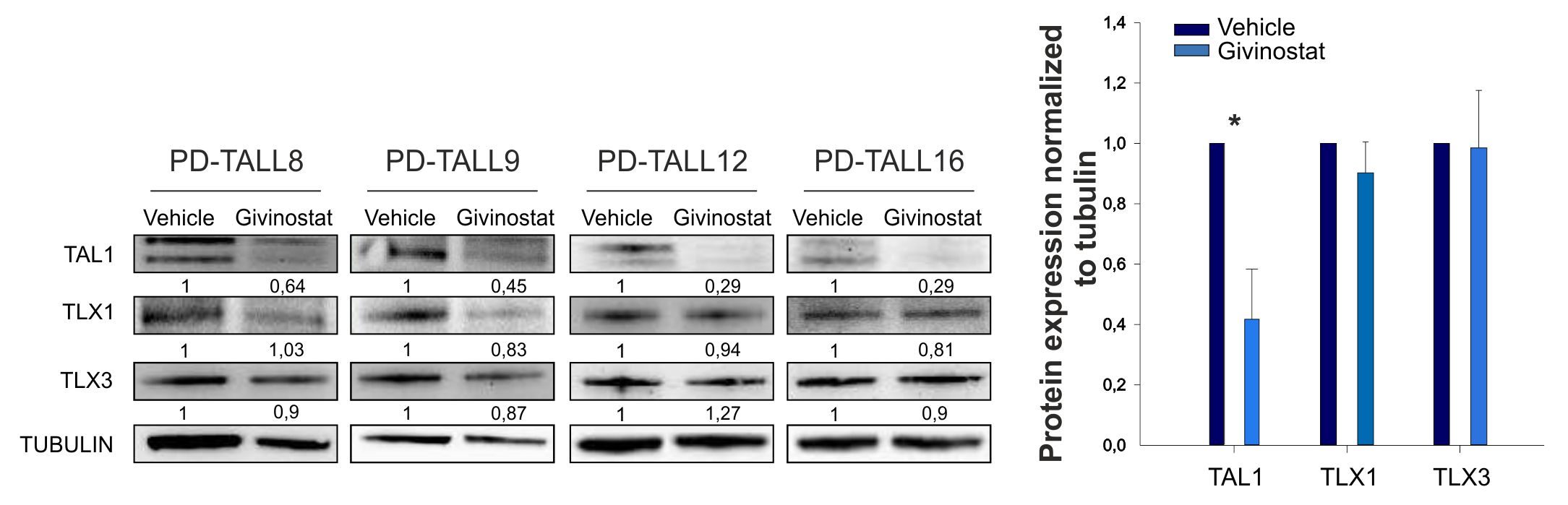
**

**Supplementary figure 1. Evaluation of TAL1, TLX1 and TLX3 protein levels in T-ALL cells from xenografts treated *in vitro* with Givinostat.**

**Left Panel**: Primary PDX cells obtained from the spleen of sick mice were treated *in vitro* for 16 h with Givinostat or vehicle and protein levels of TLX1, TLX3 and TAL1 were analyzed by western blot. Numbers below the bands indicate values of densitometric analysis of TLX1, TLX3 and TAL1 bands normalized to TUBULIN.

**Right panel**: Columns report the mean values ± s.d. of TLX1, TLX3 and TAL1 ratios in all PDX analyzed (n=4). The ratio in vehicle-treated cells was set at 1 (^*^*P*<0.05).

**Suppl. Fig. 2**

**
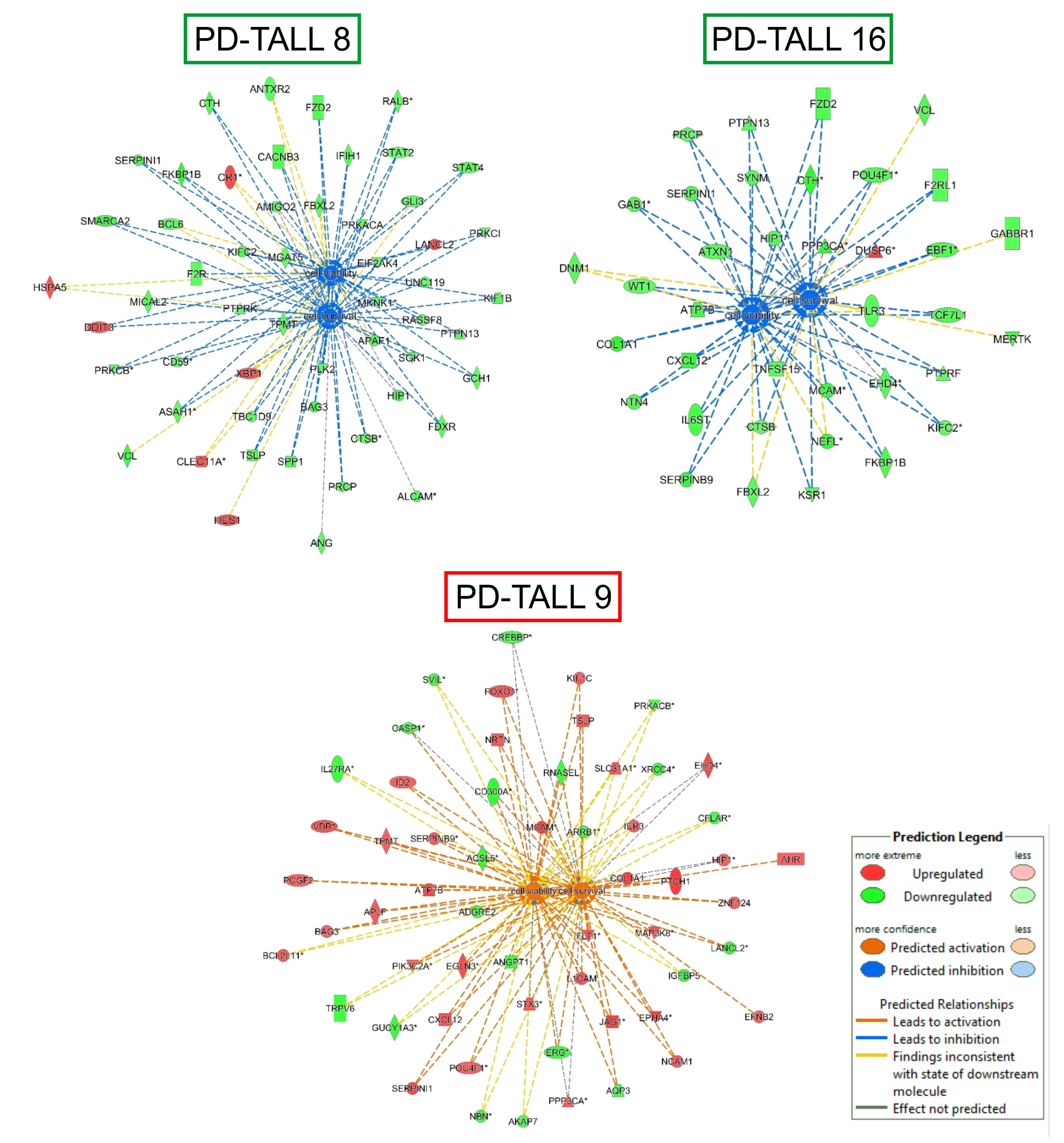
**

**Suppl. Fig. 2. Givinostat causes repression of cell survival and cell viability pathways in good responders but not in the poor responder*.*** IPA functional analysis showed the repression of gene networks related to cell survival and cell viability in the treated group of both good responders PD-TALL8 and PD-TALL16 and activation of these pathways in the treated group of the poor-responder PD-TALL9. Genes up- or down- regulated are represented in red or green, respectively. Colours of the nodes indicate the predicted activation (orange) or repression (blue) for each specific gene network.

**Suppl. Fig. 3**

**
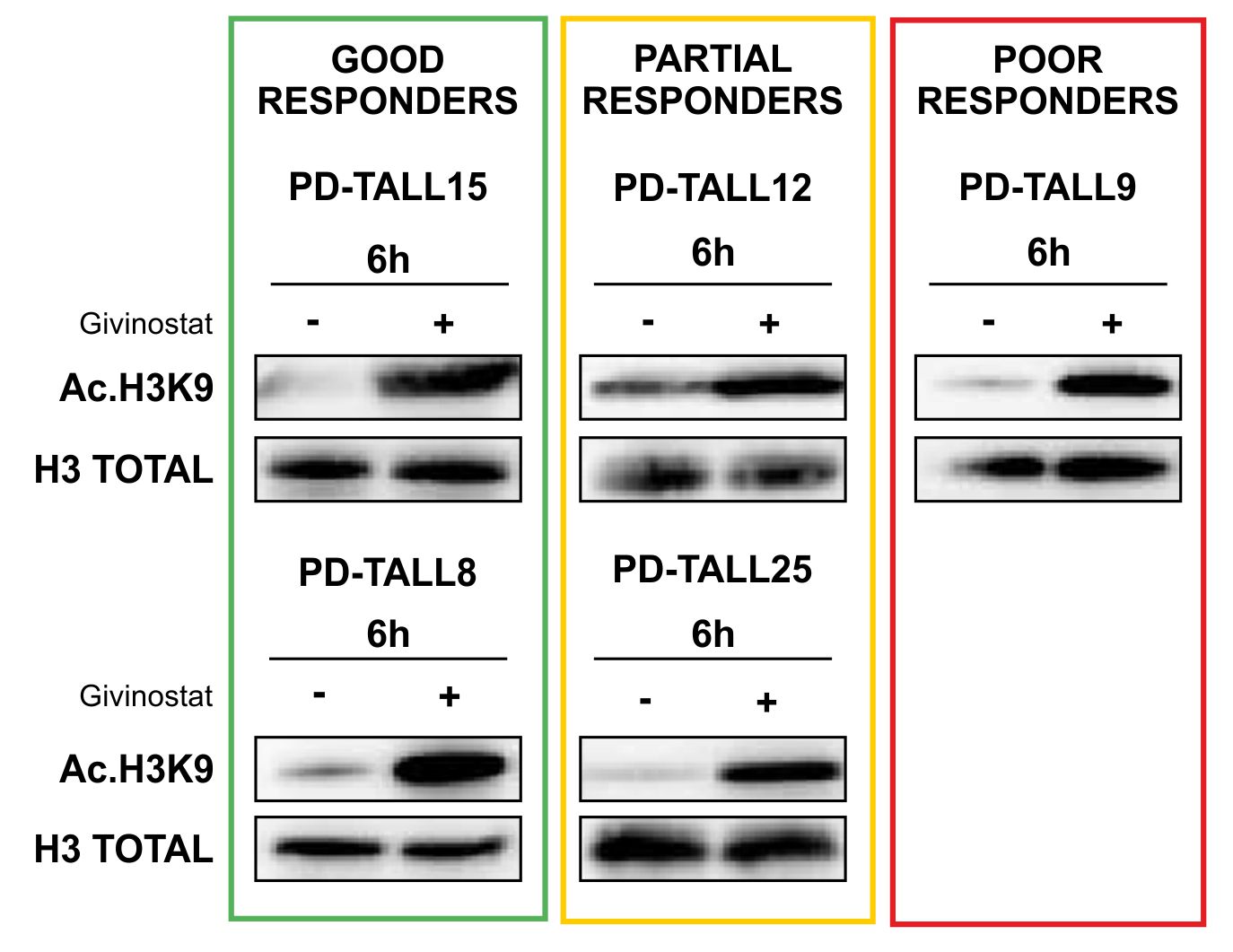
**

**Supplementary figure 3.** **Acetylation of histone H3K9 (Lysine 9) in T-ALL cells from xenografts treated *in vitro*.** Cells were recovered from the spleen of the xenografts and treated *in vitro* with Givinostat or vehicle for 6 h. Protein levels were then analyzed by western blot.

**Suppl. Figure 4**

**Supplementary figure 4: Dot plot for *HDAC6* and *SIRT2* expression in vehicle groups for each xenograft set.** At the basal level, expression levels of *HDAC6* (A) and *SIRT2* (B) are similar both in poor and good responders. The expression value for each sample (single dot) is given in a log2 scale after normalizing all gene expression data with the justRMA algorithm. X-axis represents the vehicle groups for each set of xenografts and the Y-axis represents the gene expression level.

**Suppl. Figure 5**


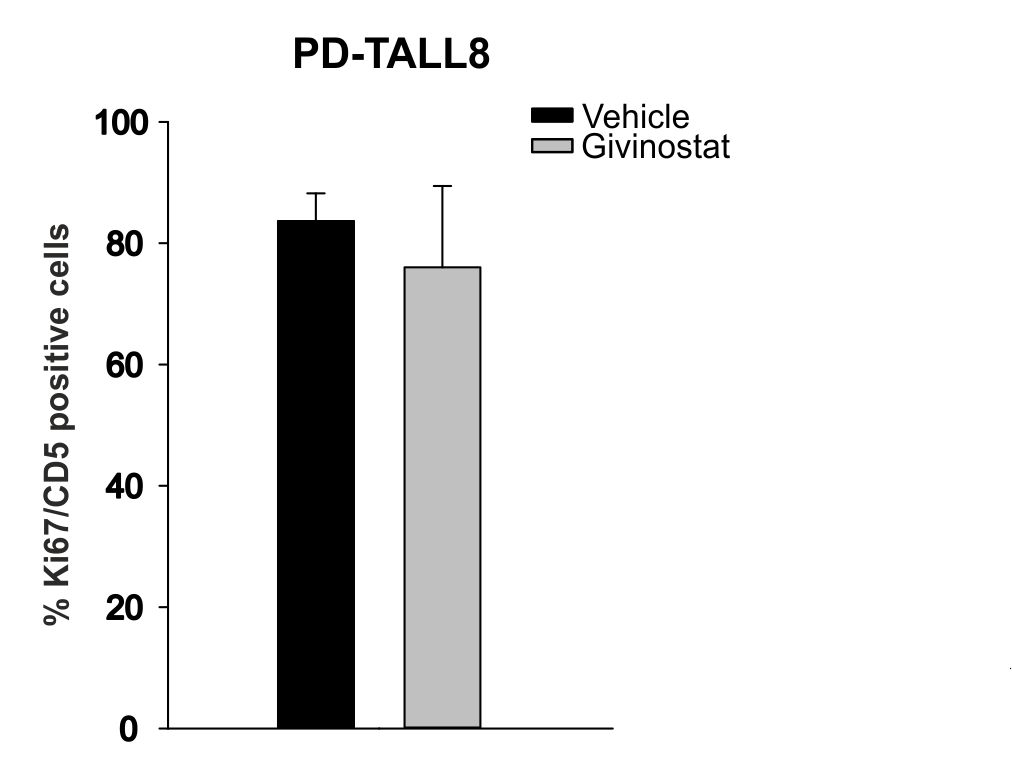


**Supplementary figure 5.** Evaluation of leukemic cell proliferation in PD-TALL8-xenografted mice treated *in vivo* with Givinostat or vehicle for five consecutive days (n=5 mice/group). Cells were recovered from the spleen and analyzed by flow cytometry following staining with CD5 and Ki67. Columns show mean ± S.D. values (n = 5 mice/group).

| **Suppl. Table I** | |  |  |
| --- | --- | --- | --- |
|  |  |  |  |
| **Primary antibodies used to analyzed** | | |  |
| **xenografts immunophenotype** | | |  |
| *Antigen* | *Reactivity* | *Conjugate* | *Company* |
| CD4 | human | FITC | BD Biosciences, San Jose, CA |
| CD8 | human | PE | BD Biosciences, San Jose, CA |
| CD34 | human | PE-Cy5 | BD Biosciences, San Jose, CA |
| CD5 | human | PE-Cy7 | Coulter, Fullerton, CA |
| CD2 | human | APC | Coulter, Fullerton, CA |
| CD3 | human | APC-Cy7 | Coulter, Fullerton, CA |
| CD7 | human | HORIZON 450 | Coulter, Fullerton, CA |
| CD45 | human | HORIZON 500 | BD Biosciences, San Jose, CA |
| CD99 | human | FITC | BD Biosciences, San Jose, CA |
| CD11b | human | PE | Coulter, Fullerton, CA |
| CD117 | human | PE-Cy7 | Coulter, Fullerton, CA |
| CD1a | human | FITC | BD Biosciences, San Jose, CA |
| CD10 | human | PE | Coulter, Fullerton, CA |
| CyCD3 | human | APC | BD Biosciences, San Jose, CA |

| **Suppl. Table II** |  |
| --- | --- |
|  |  |
| **Primers used for qPCR analysis** | |
| *hRAD50-*for | 5’-GGGAAAGACGACCATCATTG-3’ |
| *hRAD50-*rev | 5’-AGCAACCTTGGGATCGTGTA-3’ |
| *hMLH1-*for | 5’-CCCAAAGAAGGACTTGCTGA-3’ |
| *hMLH1-*rev | 5’-CAATCAGGTTCCCTTCCTCA-3’ |
| *hNBN-*for | 5’-ACAAGGCGTGTCAGTTGATG-3’ |
| *hNBN-*rev | 5’-TTGGCCTTTCACTCAAATCC-3’ |
| *hCDC73-*for | 5’-ACCAAACAGCCTATCCCAGC-3’ |
| *hCDC73-*rev | 5’-CAGATGCACCCTCCGTTACA-3’ |
| *hDLL1-*for | 5’-CTGATGACCTCGCAACAGAA-3’ |
| *hDLL1-*rev | 5’-ACACACGAAGCGGTAGGAGT-3’ |
| *hJAG1-*for | 5’-AGGGCAAGAACTGCTCACAC-3’ |
| *hJAG1-*rev | 5’-TTCAGGTGTGTCGTTGGAAG-3’ |
| *h*β*2-microglobulin-*for | 5'-TGCTGTCTCCATGTTTGATGTATCT-3' |
| *h*β*2-microglobulin-*rev | 5'-TCTCTGCTCCCCACCTCTAAGT-3' |
|  |  |
| **RealTime Ready Custom Panel Primers** | |
| *hSTAT5A*-for | 5'-GCTCCCTATAACATGTACCCACA-3' |
| *hSTAT5A*-rev | 5'-CTGGCCACATCCATGGTC-3' |
| *hTRIB2*-for | 5'-CATCTTTAAGGACGAAGAGAGGA-3' |
| *hTRIB2*-rev | 5'-TTCAAGATCTCTGGGCTTACG-3' |
| *hBMI1*-for | 5'-TGTAAAACGTGTATTGTTCGTTACC-3' |
| *hBMI1*-rev | 5'-CAATATCTTGGAGAGTTTTATCTGACC-3' |
| *hRUNX1*-for | 5'-TGCCTCCCTGAACCACTC-3' |
| *hRUNX1*-rev | 5'-GATGGTTGGATCTGCCTTGTA-3' |
| *hALDH1A1*-for | 5'-CCAAAGACATTGATAAAGCCATAA-3' |
| *hALDH1A1*-rev | 5'-CACGCCATAGCAATTCACC-3' |
| *hCCR7*-for | 5'-TGGTGGCTCTCCTTGTCATT-3' |
| *hCCR7*-rev | 5'-GCTTTAAAGTTCCGCACGTC-3' |
| *hPTPN14*-for | 5'-CAGGGAGTGAATGTGATTGC-3' |
| *hPTPN14*-rev | 5'-CAGTATCGGTGGCTTTTGGT-3' |
| *hPLXND1*-for | 5'-CCCTGAGCCCATGACAGT-3' |
| *hPLXND1*-rev | 5'-AGGCACTGGGAACAGTCG-3' |
| *hGBP5*-for | 5'-CAAATCACACATTAGTTCTGCTTGA-3' |
| *hGBP5*-rev | 5'-TGGATATCATTCTTGTTGTCAGC-3' |
| *hGATA2*-for | 5'-CACAAGATGAATGGGCAGAA-3' |
| *hGATA2*-rev | 5'-TGACAATTTGCACAACAGGTG-3' |

| **Suppl. Table III** |  |
| --- | --- |
|  |  |
| **Primary antibodies used for Western Blot analysis** |  |
|  |  |
| *Description* | *Company* |
| Mouse Ab against Acetylated α Tubulin | Santa Cruz Biotechnologies, Dallas, Texas |
| Rabbit Ab against Actin | Sigma Aldrich, Saint Luis, MO |
| Rabbit Ab against TLX1 | Sigma Aldrich, Saint Luis, MO |
| Rabbit Ab against TLX3 | Sigma Aldrich, Saint Luis, MO |
| Mouse Ab against TAL1 | Merck-Millipore, Darmstadt, Germany |
| Rabbit Ab against Acetylated Histone H3K9 | Cell Signaling Technology, Danvers, MA |
| Rabbit Ab against total Histone H3K9 | Cell Signaling Technology, Danvers, MA |
| Rabbit Ab against Phosphorylated Histone H2AX | Cell Signaling Technology, Danvers, MA |
| Rabbit Ab against total Histone H2AX | Cell Signaling Technology, Danvers, MA |

| **Suppl. Table IV**    **Patients immunophenotype at diagnosis** | | | | | | |  |  |  |
| --- | --- | --- | --- | --- | --- | --- | --- | --- | --- |
|  | | | | | | |  |  |  |
|  |  |  |  |  |  |  |  |  |  |
|  | **PD-TALL6** | **PD-TALL8** | **PD-TALL9** | **PD-TALL12** | **PD-TALL15** | **PD-TALL16** | **PD-TALL19** | **PD-TALL25** | **PD-TALL43** |
| **CD45** | POS B | POS B | POS B | POS B | POS B | POS B | POS B | POS B | POS B |
| **CD1a** | P.E. D | P.E. D | NEG | NEG | NEG | NEG | NEG | NEG | P.E. D |
| **CD2** | POS B | POS B | P.E. D | POS B | P.E. D | NEG | POS D | P.E. D | POS B |
| **CD3** | POS | P.E. D | P.E. D | NEG | P.E. D | POS D | NEG | P.E. D | P.E. D |
| **CD4** | NEG | NEG | NEG | NEG | P.E. D | NEG | POS D | POS | P.E. D |
| **CD5** | POS B | POS B | POS B | POS B | POS B | POS B | POS B | POS B | POS B |
| **CD7** | POS B | POS | POS B | POS B | POS B | POS B | POS B | POS H | POS B |
| **CD8** | POS | NEG | P.E. D | P.E. D | P.E. D | NEG | NEG | POS | POS B |
| **CYCD3** | POS B | POS B | POS B | POS B | POS B | POS | POS B | POS | POS B |
| **CD10** | NEG | NEG | NEG | NEG | NEG | NEG | ND | NEG | NEG |
| **CD19** | NEG | NEG | NEG | NEG | NEG | NEG | NEG | NEG | NEG |
| **CD20** | NEG | NEG | ND | NEG | NEG | NEG | ND | ND | ND |
| **CD52** | POS B | POS B | P.E. D | P.E. D | POS B | POS D | POS D | POS B | POS B |
| **CD44** | POS B | POS B | POS B | POS B | POS D | POS D | POS D | NEG | POS B |
| **CD16** | NEG | NEG | NEG | NEG | NEG | NEG | NEG | NEG | NEG |
| **CD56** | NEG | NEG | NEG | NEG | NEG | NEG | NEG | NEG | P.E. D |
| **CD13** | NEG | NEG | NEG | NEG | NEG | NEG | NEG | NEG | NEG |
| **CD14** | NEG | NEG | NEG | NEG | NEG | NEG | NEG | NEG | NEG |
| **CD33** | NEG | NEG | NEG | NEG | NEG | P.E. D | NEG | NEG | NEG |
| **CD65** | NEG | NEG | NEG | NEG | NEG | NEG | NEG | NEG | NEG |
| **CD38** | POS B | POS B | POS B | POS B | POS B | POS B | POS B | POS B | POS B |
| **CD34** | P.E. H | NEG | P.E. D | P.E. H | NEG | NEG | NEG | NEG | NEG |
| **CD117** | NEG | P.E D | P.E. D | NEG | NEG | NEG | NEG | NEG | NEG |
| **CD135** | NEG | NEG | NEG | NEG | NEG | NEG | NEG | P.E. D | POS D |
| **CD25** | NEG | NEG | NEG | NEG | NEG | NEG | NEG | NEG | NEG |
| **CD99** | POS B | POS B | ND | ND | POS B | NEG | POS B | POS D | POS B |
| **POS** = Positive; **NEG** = Negative; **P.E**.= Partially Expressed; **D**.= Dim; **B.** = Bright; **H.** = Heterogeneous; **ND** = Not determined | | | | | | | | | |

|  |  |  |  |  |  |  |
| --- | --- | --- | --- | --- | --- | --- |

**Suppl. Table V**

| **UP-REGULATED IN GOOD RESPONDERS (GIV8 and GIV16)** | | | | |  |  |
| --- | --- | --- | --- | --- | --- | --- |
| **GIV8 vs GIV9** |  |  |  | **GIV16vsGIV9** |  |  |
| **Gene Symbol** | **Probe-set** | **Fold Change** |  | **Gene Symbol** | **Probe-set** | **Fold Change** |
| LOC100506451 | 242358_at | 2.07 |  | MAML2 | 235457_at | 1.78 |
| C6orf147 | 241024_at | 1.65 |  | RGS5 | 230678_at | 1.60 |
| CTPS2 | 222819_at | 1.64 |  | IFIT1 | 203153_at | 1.56 |
| VPREB1 | 221349_at | 1.60 |  | EOGT | 221935_s_at | 1.53 |
| LOC257396 | 236076_at | 1.60 |  | CTPS2 | 222819_at | 1.52 |
| RGS5 | 230678_at | 1.60 |  | EPSTI1 | 227609_at | 1.52 |
| LOC400680 | 231470_at | 1.58 |  | C1RL | 218983_at | 1.51 |
| EOGT | 221935_s_at | 1.56 |  | PPAP2B | 212230_at | 1.50 |
| CNTLN | 220095_at | 1.53 |  | DNAJC3-AS1 | 240574_at | 1.49 |
| MAML2 | 235457_at | 1.53 |  | SAMD13 | 229402_at | 1.47 |
| NEDD4 | 213012_at | 1.48 |  | TRAM1L1 | 244334_at | 1.47 |
| C1RL | 218983_at | 1.48 |  | ADAMTS3 | 214913_at | 1.47 |
| NUDT9P1 | 1552564_at | 1.48 |  | BZRAP1-AS1 | 228826_at | 1.47 |
| KIAA0240 | 213208_at | 1.47 |  | LOC100506451 | 242358_at | 1.47 |
| VRK2 | 205126_at | 1.46 |  | HIST1H2BO | 214540_at | 1.46 |
| CSRP2 | 207030_s_at | 1.45 |  | LOC400680 | 231470_at | 1.46 |
| LPGAT1 | 1555058_a_at | 1.45 |  | CSRP2 | 207030_s_at | 1.45 |
| KIAA1107 | 214098_at | 1.45 |  | XRCC4 | 205072_s_at | 1.45 |
| C5orf42 | 219381_at | 1.44 |  | GRB10 | 209409_at | 1.44 |
| HDGFRP3 | 228266_s_at | 1.44 |  | C5orf42 | 219381_at | 1.43 |
| IFIT1 | 203153_at | 1.43 |  | RLN1 | 211753_s_at | 1.42 |
| LOC728613 | 1569110_x_at | 1.40 |  | CTSO | 203758_at | 1.42 |
| RLN1 | 211753_s_at | 1.40 |  | NUDT7 | 228855_at | 1.41 |
| DNAJC3-AS1 | 240574_at | 1.39 |  | C2CD2 | 212875_s_at | 1.41 |
| LOC100131662 | 236973_at | 1.39 |  | C6orf147 | 241024_at | 1.41 |
| ERAP1 | 214012_at | 1.39 |  | LOC100131662 | 236973_at | 1.41 |
| WARS2 | 222734_at | 1.39 |  | KIAA0240 | 213208_at | 1.40 |
| CD96 | 1555120_at | 1.37 |  | AKAP7 | 205771_s_at | 1.39 |
| CTSO | 203758_at | 1.37 |  | NUDT9P1 | 1552564_at | 1.39 |
| PPAP2B | 212230_at | 1.37 |  | CNTLN | 1559005_s_at | 1.39 |
| NUDT7 | 228855_at | 1.36 |  | NEDD4 | 213012_at | 1.38 |
| GPR137B | 204137_at | 1.35 |  | CFLAR | 235427_at | 1.38 |
| PIF1 | 228252_at | 1.35 |  | CTH | 217127_at | 1.38 |
| DENND5B | 228551_at | 1.35 |  | LINC00662 | 1558256_at | 1.38 |
| TSC22D1 | 235315_at | 1.35 |  | KIAA1107 | 214098_at | 1.36 |
| BZRAP1-AS1 | 228826_at | 1.34 |  | LOC728613 | 1569110_x_at | 1.36 |
| ZEB1-AS1 | 229090_at | 1.34 |  | LPGAT1 | 1555058_a_at | 1.36 |
| PRKD3 | 218236_s_at | 1.34 |  | LOC257396 | 236076_at | 1.35 |
| ZNF678 | 242923_at | 1.34 |  | APOL3 | 221087_s_at | 1.35 |
| LOC100130000 | 230837_at | 1.34 |  | CD96 | 1555120_at | 1.34 |
| ADAMTS3 | 214913_at | 1.33 |  | DET1 | 219641_at | 1.34 |
| C2CD2 | 212875_s_at | 1.33 |  | NAGPA | 205090_s_at | 1.33 |
| KLHDC5 | 225961_at | 1.33 |  | COX15 | 223281_s_at | 1.33 |
| SAMD13 | 229402_at | 1.33 |  | KDELC2 | 225128_at | 1.33 |
| TRAM1L1 | 244334_at | 1.33 |  | LOC100130000 | 230837_at | 1.33 |
| HIST1H2BO | 214540_at | 1.32 |  | SIDT1 | 219734_at | 1.33 |
| XRCC4 | 210813_s_at | 1.32 |  | PVRIG | 219812_at | 1.33 |
| DET1 | 219641_at | 1.32 |  | PDSS2 | 1555886_at | 1.32 |
| SIDT1 | 219734_at | 1.32 |  | ZNF678 | 242923_at | 1.32 |
| SMAD1 | 210993_s_at | 1.31 |  | KIAA0141 | 201977_s_at | 1.32 |
| KDELC2 | 225128_at | 1.31 |  | AFAP1L2 | 226829_at | 1.32 |
| TTC7B | 226152_at | 1.31 |  | ZAK | 223519_at | 1.32 |
| DZIP3 | 207232_s_at | 1.31 |  | ADCY7 | 203741_s_at | 1.31 |
| NBR2 | 1553992_s_at | 1.30 |  | MLKL | 238025_at | 1.31 |
| TBCK | 226126_at | 1.30 |  | TSC22D1 | 235315_at | 1.30 |
| ADCY7 | 203741_s_at | 1.30 |  | METTL25 | 229018_at | 1.30 |
| DLGAP1-AS1 | 238432_at | 1.30 |  | VRK2 | 205126_at | 1.30 |
| AKAP7 | 205771_s_at | 1.29 |  | LINC00476 | 239799_at | 1.30 |
| ZHX2 | 203556_at | 1.29 |  | DDX60 | 218986_s_at | 1.30 |
| FAAH2 | 230792_at | 1.29 |  | GPR137B | 204137_at | 1.30 |
| COX15 | 223281_s_at | 1.29 |  | LOC100996286 | 1559205_s_at | 1.30 |
| NAGPA | 205090_s_at | 1.28 |  | KLHDC5 | 225961_at | 1.29 |
| LNPEP | 236728_at | 1.28 |  | FBXO4 | 223493_at | 1.29 |
| ZAK | 223519_at | 1.28 |  | ZEB1-AS1 | 229090_at | 1.29 |
| RNASEH2B | 229210_at | 1.28 |  | VWA8 | 214231_s_at | 1.29 |
| C11orf71 | 218789_s_at | 1.28 |  | ZHX2 | 203556_at | 1.29 |
| GINS3 | 218719_s_at | 1.28 |  | C19orf54 | 222052_at | 1.28 |
| PVRIG | 219812_at | 1.28 |  | PIGK | 209707_at | 1.28 |
| LOC100996286 | 1559205_s_at | 1.28 |  | PHF15 | 212660_at | 1.28 |
| PIGK | 209707_at | 1.27 |  | SPATA5 | 229075_at | 1.28 |
| EPSTI1 | 227609_at | 1.27 |  | WARS2 | 218766_s_at | 1.27 |
| FBXO4 | 223493_at | 1.27 |  | NIPSNAP3A | 224436_s_at | 1.27 |
| C19orf54 | 222052_at | 1.27 |  | C11orf71 | 218789_s_at | 1.27 |
| DDX60 | 218986_s_at | 1.27 |  | ELK3 | 221773_at | 1.26 |
| PEX5L | 222910_s_at | 1.26 |  | TBCK | 226126_at | 1.26 |
| LINC00662 | 1558256_at | 1.26 |  | DLGAP1-AS1 | 238432_at | 1.26 |
| FKRP | 219853_at | 1.26 |  | ZFX | 229022_at | 1.26 |
| MLKL | 238025_at | 1.26 |  | PIGV | 51146_at | 1.26 |
| GRB10 | 209410_s_at | 1.26 |  | LNPEP | 236728_at | 1.26 |
| ZFX | 207920_x_at | 1.25 |  | ISG20 | 204698_at | 1.25 |
| AFAP1L2 | 226829_at | 1.25 |  | NCOA2 | 212867_at | 1.25 |
| APOL3 | 221087_s_at | 1.25 |  | RAD50 | 208393_s_at | 1.25 |
| LOC100996578 | 235134_at | 1.25 |  | AZI2 | 227904_at | 1.25 |
| SMARCAL1 | 218452_at | 1.24 |  | GINS3 | 218719_s_at | 1.24 |
| ZMYND11 | 202136_at | 1.23 |  | PIK3CG | 206369_s_at | 1.24 |
| ZBTB2 | 214482_at | 1.23 |  | MON1B | 233557_s_at | 1.24 |
| PTPMT1 | 225901_at | 1.23 |  | SLAMF6 | 1552497_a_at | 1.24 |
| PIK3CG | 206369_s_at | 1.23 |  | PIF1 | 228252_at | 1.23 |
| TRMT11 | 218877_s_at | 1.23 |  | SMAD1 | 210993_s_at | 1.23 |
| HAUS4 | 218383_at | 1.23 |  | RNASEH2B | 229210_at | 1.23 |
| LOC257152 | 215302_at | 1.23 |  | ATXN7L1 | 227732_at | 1.23 |
| LOC100996579 | 236598_at | 1.23 |  | DENND5B | 238917_s_at | 1.23 |
| NCOA2 | 212867_at | 1.23 |  | TTC7B | 226152_at | 1.23 |
| RBM23 | 219816_s_at | 1.23 |  | LOC100996579 | 236598_at | 1.23 |
| RCSD1 | 225763_at | 1.22 |  | AP2B1 | 200615_s_at | 1.23 |
| ELK3 | 221773_at | 1.22 |  | TRMT11 | 218877_s_at | 1.22 |
| ZC3H4 | 213390_at | 1.22 |  | CDC73 | 235196_at | 1.22 |
| RAD50 | 208393_s_at | 1.22 |  | MYO1B | 212364_at | 1.22 |
| CFLAR | 210563_x_at | 1.22 |  | PRKD3 | 218236_s_at | 1.22 |
| MYO1B | 212364_at | 1.22 |  | ZNF415 | 205514_at | 1.22 |
| OXCT1 | 202780_at | 1.22 |  | LOC100996578 | 235134_at | 1.22 |
| YTHDC2 | 1568680_s_at | 1.22 |  | ARHGAP21 | 224764_at | 1.22 |
| HELLS | 227349_at | 1.22 |  | NBR2 | 1553992_s_at | 1.22 |
| METTL25 | 229018_at | 1.22 |  | ZFP1 | 226807_at | 1.21 |
| ERBB2IP | 222473_s_at | 1.21 |  | CCDC111 | 227157_at | 1.21 |
| ISG20 | 204698_at | 1.21 |  | FKRP | 227882_at | 1.21 |
| ZNF415 | 205514_at | 1.20 |  | HDGFRP3 | 209526_s_at | 1.21 |
| RAB37 | 228113_at | 1.20 |  | ZC3H4 | 213390_at | 1.21 |
| LINC00476 | 227893_at | 1.20 |  | VPREB1 | 221349_at | 1.21 |
| GLMN | 207153_s_at | 1.20 |  | FAAH2 | 230792_at | 1.20 |
| PHF15 | 212660_at | 1.20 |  | LOC257152 | 215302_at | 1.20 |
| DBF4B | 238508_at | 1.20 |  | SPC25 | 209891_at | 1.20 |
| SPATA5 | 229075_at | 1.20 |  | PLSCR1 | 202430_s_at | 1.20 |
| ZFYVE16 | 203651_at | 1.20 |  | CENPN | 222118_at | 1.20 |
| LOC728769 | 238039_at | 1.19 |  | GLMN | 207153_s_at | 1.20 |
| NIPSNAP3A | 224436_s_at | 1.19 |  | VOPP1 | 208091_s_at | 1.19 |
| KIAA0182 | 212057_at | 1.19 |  | NBN | 202907_s_at | 1.19 |
| SPC25 | 209891_at | 1.19 |  | PEX5L | 222910_s_at | 1.19 |
| ATXN7L1 | 232265_at | 1.19 |  | AGL | 203566_s_at | 1.19 |
| HAUS5 | 213054_at | 1.19 |  | HMMR | 209709_s_at | 1.19 |
| AZI2 | 227904_at | 1.19 |  | NFYC | 202215_s_at | 1.19 |
| DARS | 218365_s_at | 1.19 |  | RNLS | 223824_at | 1.19 |
| CDC73 | 235196_at | 1.19 |  | DZIP3 | 213186_at | 1.19 |
| MICA | 221779_at | 1.19 |  | NMI | 203964_at | 1.19 |
| MICALL1 | 221779_at | 1.19 |  | DBF4B | 238508_at | 1.19 |
| DCTD | 201572_x_at | 1.18 |  | MICA | 221779_at | 1.18 |
| PDSS2 | 219307_at | 1.18 |  | MICALL1 | 221779_at | 1.18 |
| DEPDC1B | 226980_at | 1.18 |  | LOC728769 | 238039_at | 1.18 |
| PIGV | 219238_at | 1.18 |  | OXCT1 | 202780_at | 1.18 |
| RNLS | 223824_at | 1.18 |  | KIAA0182 | 212057_at | 1.18 |
| AGL | 203566_s_at | 1.18 |  | RBM23 | 219816_s_at | 1.18 |
| HMMR | 209709_s_at | 1.18 |  | DEPDC1B | 226980_at | 1.18 |
| TAF9B | 226037_s_at | 1.18 |  | PTS | 209694_at | 1.17 |
| AP2B1 | 200615_s_at | 1.18 |  | HAUS5 | 213054_at | 1.17 |
| MON1B | 233557_s_at | 1.18 |  | ZMYND11 | 202136_at | 1.17 |
| TBC1D1 | 227945_at | 1.17 |  | RAB37 | 228113_at | 1.17 |
| MEPCE | 219798_s_at | 1.17 |  | YTHDC2 | 213077_at | 1.17 |
| PPIE | 210502_s_at | 1.17 |  | RCSD1 | 225763_at | 1.17 |
| CENPN | 228559_at | 1.17 |  | ASIC1 | 205156_s_at | 1.16 |
| TIGD1 | 1553099_at | 1.16 |  | PTPMT1 | 225901_at | 1.16 |
| NBN | 202906_s_at | 1.16 |  | MEPCE | 219798_s_at | 1.16 |
| SETDB1 | 203155_at | 1.16 |  | LOC100506100 | 228773_at | 1.16 |
| POT1 | 204353_s_at | 1.16 |  | TAF9B | 226037_s_at | 1.16 |
| NFYC | 211797_s_at | 1.16 |  | TBC1D1 | 227945_at | 1.16 |
| C15orf44 | 221265_s_at | 1.16 |  | SMARCAL1 | 218452_at | 1.15 |
| ZFP1 | 226807_at | 1.15 |  | R3HCC1 | 212866_at | 1.15 |
| ARHGAP21 | 224764_at | 1.15 |  | PPIE | 210502_s_at | 1.15 |
| SLAMF6 | 1552497_a_at | 1.15 |  | C15orf44 | 221265_s_at | 1.15 |
| VOPP1 | 208091_s_at | 1.15 |  | MLH1 | 202520_s_at | 1.15 |
| CCDC111 | 227157_at | 1.15 |  | ZBTB2 | 226284_at | 1.15 |
| NMI | 203964_at | 1.15 |  | ZFYVE16 | 203651_at | 1.15 |
| MLH1 | 202520_s_at | 1.15 |  | TIGD1 | 1553099_at | 1.15 |
| LCMT2 | 204012_s_at | 1.15 |  | HELLS | 223556_at | 1.14 |
| CDC16 | 202717_s_at | 1.15 |  | HAUS4 | 218383_at | 1.14 |
| R3HCC1 | 212866_at | 1.15 |  | SETDB1 | 203155_at | 1.14 |
| VWA8 | 212946_at | 1.15 |  | CDC16 | 202717_s_at | 1.14 |
| BRD7 | 222737_s_at | 1.15 |  | SLC41A3 | 224931_at | 1.13 |
| PTS | 209694_at | 1.15 |  | ERBB2IP | 217941_s_at | 1.13 |
| PLK1S1 | 219961_s_at | 1.14 |  | MRPS27 | 212145_at | 1.13 |
| LOC100506100 | 228773_at | 1.14 |  | CTCF | 202521_at | 1.13 |
| BUB3 | 201456_s_at | 1.14 |  | MRPS33 | 218654_s_at | 1.13 |
| SLC41A3 | 219175_s_at | 1.14 |  | SREK1 | 212721_at | 1.13 |
| CDC7 | 204510_at | 1.14 |  | PLK1S1 | 219961_s_at | 1.12 |
| ASIC1 | 205156_s_at | 1.14 |  | POT1 | 204354_at | 1.12 |
| CEP170 | 207719_x_at | 1.14 |  | SEPHS1 | 208941_s_at | 1.12 |
| SREK1 | 212721_at | 1.14 |  | ERAP1 | 209788_s_at | 1.12 |
| RRM2 | 209773_s_at | 1.13 |  | ZCCHC9 | 225538_at | 1.12 |
| FBXO25 | 225591_at | 1.13 |  | DCTD | 201572_x_at | 1.12 |
| CTCF | 202521_at | 1.13 |  | CDC7 | 204510_at | 1.12 |
| CTH | 202521_at | 1.13 |  | LCMT2 | 204012_s_at | 1.12 |
| PLSCR1 | 202446_s_at | 1.12 |  | FBXO25 | 225591_at | 1.12 |
| MRPS33 | 218654_s_at | 1.12 |  | TSPAN7 | 202242_at | 1.12 |
| KIAA0141 | 201978_s_at | 1.12 |  | NDUFB5 | 203621_at | 1.11 |
| MPHOSPH6 | 203740_at | 1.12 |  | MPHOSPH6 | 203740_at | 1.11 |
| SEPHS1 | 208941_s_at | 1.12 |  | BRD7 | 221776_s_at | 1.10 |
| ZCCHC9 | 225538_at | 1.12 |  | CNIH | 201653_at | 1.10 |
| MRPS27 | 212145_at | 1.12 |  | PNISR | 212176_at | 1.10 |
| CNIH | 201653_at | 1.10 |  | BUB3 | 201458_s_at | 1.10 |
| CBFB | 202370_s_at | 1.10 |  | RRM2 | 201890_at | 1.10 |
| PNISR | 212176_at | 1.10 |  | CBFB | 202370_s_at | 1.10 |
| NDUFB5 | 203621_at | 1.09 |  | CEP170 | 207719_x_at | 1.10 |
| UBE2E1 | 212519_at | 1.09 |  | UBE2E1 | 212519_at | 1.10 |
| TSPAN7 | 202242_at | 1.09 |  | DARS | 201623_s_at | 1.08 |
|  |  |  |  |  |  |  |
| **UP-REGULATED IN POOR RESPONDER (GIV9)** | | | |  |  |  |
| **GIV8 vs GIV9** |  |  |  | **GIV16vsGIV9** |  |  |
| **Gene Symbol** | **Probe-set** | **Fold Change** |  | **Gene Symbol** | **Probe-set** | **Fold Change** |
| C20orf24 | 223880_x_at | 1.07 |  | PSMG1 | 203405_at | 1.08 |
| FAM32A | 201863_at | 1.08 |  | C20orf24 | 223880_x_at | 1.08 |
| MTERFD1 | 219363_s_at | 1.10 |  | TMED2 | 200087_s_at | 1.08 |
| SPSB3 | 46256_at | 1.10 |  | SLC9A6 | 203909_at | 1.08 |
| CSGALNACT2 | 222235_s_at | 1.10 |  | SPSB3 | 46256_at | 1.09 |
| NIPA2 | 212129_at | 1.10 |  | MTERFD1 | 219363_s_at | 1.09 |
| VPS26A | 201807_at | 1.10 |  | RAPGEF6 | 230078_at | 1.10 |
| TMED2 | 200087_s_at | 1.11 |  | PLEKHB2 | 201410_at | 1.10 |
| SNHG15 | 225699_at | 1.11 |  | SHOC2 | 202777_at | 1.10 |
| NANS | 218189_s_at | 1.11 |  | CSGALNACT2 | 222235_s_at | 1.10 |
| TMEM50A | 222401_s_at | 1.11 |  | TM2D2 | 224413_s_at | 1.11 |
| IRGQ | 64488_at | 1.11 |  | ZFP36L1 | 211962_s_at | 1.11 |
| PSMG1 | 203405_at | 1.11 |  | EIF5B | 201024_x_at | 1.11 |
| ZFP36L1 | 211962_s_at | 1.12 |  | IRGQ | 221877_at | 1.11 |
| MANF | 202655_at | 1.12 |  | C11orf82 | 228281_at | 1.11 |
| MAPK8IP3 | 213178_s_at | 1.12 |  | VPS26A | 201807_at | 1.11 |
| H2AFJ | 224301_x_at | 1.12 |  | TMEM50A | 217766_s_at | 1.11 |
| IDH1 | 201193_at | 1.12 |  | MANF | 202655_at | 1.12 |
| SHOC2 | 202777_at | 1.12 |  | TSEN34 | 218132_s_at | 1.12 |
| DUSP1 | 201041_s_at | 1.12 |  | GATAD2A | 222526_at | 1.12 |
| RAPGEF6 | 230078_at | 1.13 |  | NDE1 | 218414_s_at | 1.12 |
| GATAD2A | 222526_at | 1.13 |  | SNHG15 | 225699_at | 1.12 |
| DCTPP1 | 218069_at | 1.13 |  | NANS | 218189_s_at | 1.12 |
| PHTF2 | 209780_at | 1.13 |  | JMJD6 | 212723_at | 1.12 |
| NDE1 | 218414_s_at | 1.14 |  | NIPA2 | 212129_at | 1.13 |
| PLEKHB2 | 201410_at | 1.14 |  | FAM32A | 201863_at | 1.13 |
| C11orf82 | 228281_at | 1.14 |  | IDH1 | 1555037_a_at | 1.13 |
| ATP6V0A2 | 205704_s_at | 1.14 |  | IER3 | 1555037_a_at | 1.13 |
| JMJD6 | 212723_at | 1.14 |  | VIMP | 223209_s_at | 1.13 |
| SLC9A6 | 203909_at | 1.14 |  | SLC35E1 | 229410_at | 1.13 |
| BCAP31 | 200837_at | 1.15 |  | TMEM184B | 202027_at | 1.13 |
| WDR60 | 219251_s_at | 1.15 |  | BCAP31 | 200837_at | 1.13 |
| VIMP | 223209_s_at | 1.15 |  | DUSP1 | 201041_s_at | 1.13 |
| FOXO6 | 239657_x_at | 1.15 |  | CYB5R1 | 202263_at | 1.13 |
| RIT1 | 209882_at | 1.16 |  | MAPK8IP3 | 213178_s_at | 1.14 |
| ZNF791 | 1553704_x_at | 1.16 |  | PHTF2 | 209780_at | 1.14 |
| CRAMP1L | 225172_at | 1.16 |  | SBNO1 | 229528_at | 1.14 |
| TM2D2 | 224413_s_at | 1.16 |  | ERI1 | 226416_at | 1.14 |
| TMEM184B | 202027_at | 1.16 |  | PCYOX1 | 225274_at | 1.15 |
| SBNO1 | 218737_at | 1.16 |  | FNIP1 | 228768_at | 1.15 |
| SNN | 218032_at | 1.16 |  | KLHL24 | 221985_at | 1.15 |
| EIF5B | 214314_s_at | 1.16 |  | SLC25A44 | 32091_at | 1.15 |
| LAPTM4A | 200673_at | 1.16 |  | NTNG2 | 233072_at | 1.16 |
| KLHL24 | 226158_at | 1.16 |  | CCDC47 | 222432_s_at | 1.16 |
| FUCA1 | 202838_at | 1.17 |  | CRAMP1L | 225172_at | 1.16 |
| TSEN34 | 218132_s_at | 1.17 |  | GABRQ | 238123_at | 1.16 |
| FNIP1 | 228768_at | 1.17 |  | DCTPP1 | 218069_at | 1.16 |
| ERI1 | 226416_at | 1.17 |  | CHKA | 204266_s_at | 1.16 |
| EEF2K | 225546_at | 1.18 |  | MLXIP | 202519_at | 1.17 |
| CCDC47 | 222432_s_at | 1.18 |  | ZNF267 | 219540_at | 1.17 |
| CHKA | 204266_s_at | 1.18 |  | SNN | 218032_at | 1.17 |
| CDKN1A | 202284_s_at | 1.19 |  | FRMD8 | 227964_at | 1.17 |
| MLXIP | 225157_at | 1.19 |  | RIT1 | 209882_at | 1.17 |
| PCYOX1 | 225274_at | 1.19 |  | CDKN1A | 202284_s_at | 1.18 |
| ZNF267 | 219540_at | 1.19 |  | EFNA4 | 205107_s_at | 1.18 |
| FRMD8 | 227964_at | 1.20 |  | EEF2K | 225546_at | 1.18 |
| RAB40C | 227698_s_at | 1.22 |  | ELAVL1 | 244660_at | 1.19 |
| EPG5 | 232030_at | 1.22 |  | WDR60 | 219251_s_at | 1.20 |
| SLC25A44 | 32091_at | 1.22 |  | FLCN | 1552664_at | 1.21 |
| GABRQ | 238123_at | 1.23 |  | H2AFJ | 225245_x_at | 1.21 |
| FLT1 | 226497_s_at | 1.23 |  | FUCA1 | 202838_at | 1.21 |
| PRRT2 | 227192_at | 1.23 |  | LAPTM4A | 200673_at | 1.21 |
| MIPEPP3 | 236837_x_at | 1.24 |  | RAB40C | 227698_s_at | 1.21 |
| SLC31A1 | 203971_at | 1.24 |  | ZNF791 | 1553704_x_at | 1.21 |
| CYB5R1 | 202263_at | 1.24 |  | CYP51A1 | 202314_at | 1.21 |
| SLC35E1 | 227518_at | 1.25 |  | EGR1 | 201694_s_at | 1.21 |
| EFNA4 | 205107_s_at | 1.26 |  | MTHFR | 239035_at | 1.22 |
| AIM1L | 220289_s_at | 1.26 |  | SDE2 | 1553338_at | 1.22 |
| SDE2 | 1553338_at | 1.26 |  | FOXO6 | 239657_x_at | 1.22 |
| STARD9 | 227108_at | 1.27 |  | ATP6V0A2 | 229572_at | 1.23 |
| C10orf47 | 229801_at | 1.27 |  | ROBO3 | 219550_at | 1.23 |
| MTHFR | 239035_at | 1.28 |  | C10orf47 | 229801_at | 1.25 |
| IER3 | 201631_s_at | 1.28 |  | RABL5 | 218785_s_at | 1.25 |
| NTNG2 | 233072_at | 1.30 |  | NRTN | 210683_at | 1.25 |
| S1PR5 | 230464_at | 1.31 |  | CCDC112 | 235208_at | 1.25 |
| SV2C | 243139_at | 1.32 |  | C15orf57 | 1560814_a_at | 1.26 |
| RABL5 | 218785_s_at | 1.32 |  | MAMLD1 | 205088_at | 1.26 |
| ZCCHC14 | 212655_at | 1.32 |  | SLC31A1 | 236217_at | 1.27 |
| TTC29 | 223962_at | 1.34 |  | PRRT2 | 227192_at | 1.27 |
| NLRP3 | 207075_at | 1.35 |  | AIM1L | 220289_s_at | 1.27 |
| FLCN | 1552664_at | 1.36 |  | MIPEPP3 | 240436_at | 1.27 |
| ELAVL1 | 244660_at | 1.36 |  | NLRP3 | 207075_at | 1.29 |
| ROBO3 | 219550_at | 1.37 |  | EPG5 | 232030_at | 1.30 |
| C15orf57 | 1560814_a_at | 1.37 |  | S1PR5 | 230464_at | 1.31 |
| RASD1 | 223467_at | 1.38 |  | FLT1 | 226498_at | 1.32 |
| LINC00189 | 1553608_a_at | 1.38 |  | RASD1 | 223467_at | 1.33 |
| NFE2L3 | 236471_at | 1.39 |  | KIF5C | 203130_s_at | 1.33 |
| CYP51A1 | 216607_s_at | 1.40 |  | FOS | 209189_at | 1.35 |
| SMARCA1 | 203874_s_at | 1.40 |  | NCRNA00185 | 207063_at | 1.35 |
| AACSP1 | 1570020_at | 1.42 |  | STARD9 | 227108_at | 1.37 |
| TPBG | 203476_at | 1.43 |  | SV2C | 216086_at | 1.38 |
| TNFRSF19 | 227812_at | 1.43 |  | TPBG | 203476_at | 1.39 |
| MAMLD1 | 205088_at | 1.44 |  | TTC29 | 223962_at | 1.40 |
| EGR1 | 201693_s_at | 1.45 |  | NFE2L3 | 204702_s_at | 1.40 |
| KIF5C | 203130_s_at | 1.46 |  | SMARCA1 | 203874_s_at | 1.41 |
| NCRNA00185 | 207063_at | 1.47 |  | DLL1 | 224215_s_at | 1.42 |
| VDR | 204255_s_at | 1.50 |  | LSR | 208190_s_at | 1.42 |
| GCM1 | 206269_at | 1.50 |  | LINC00189 | 1553608_a_at | 1.42 |
| MIR22HG | 214696_at | 1.52 |  | TRIM36 | 219736_at | 1.43 |
| JAG1 | 209099_x_at | 1.52 |  | AACSP1 | 1570020_at | 1.44 |
| CCDC112 | 235208_at | 1.53 |  | MIR22HG | 214696_at | 1.44 |
| BEX5 | 229963_at | 1.57 |  | VDR | 204254_s_at | 1.48 |
| LSR | 208190_s_at | 1.62 |  | GCM1 | 206269_at | 1.49 |
| FOS | 209189_at | 1.68 |  | JAG1 | 216268_s_at | 1.50 |
| TRIM36 | 219736_at | 1.76 |  | ZCCHC14 | 212655_at | 1.52 |
| DLL1 | 224215_s_at | 1.86 |  | BEX5 | 229963_at | 1.67 |
| AHR | 202820_at | 1.88 |  | TNFRSF19 | 227812_at | 1.72 |
| NRTN | 210683_at | 1.91 |  | AHR | 202820_at | 1.85 |
|  |  |  |  |  |  |  |
|  |  |  |  |  |  |  |
